# Lentivirus-mediated RNA interference targeting *FAMLF-1* inhibits cell growth and enhances cell differentiation of acute myeloid leukemia partially differentiated cells via inhibition of AKT and c-MYC

**DOI:** 10.18632/oncotarget.21276

**Published:** 2017-09-26

**Authors:** Yuan-Mao Huang, Yi Zheng, Jing-Gang Li, Xue-Chun Wang, Ze-Chuan Wang, Wan-Ling Chen, Li-Li Pan, Yang Li, Dong-Feng Luo, Shao-Yuan Wang

**Affiliations:** ^1^ Union Clinical Medical College, Fujian Medical University, Fuzhou 350001, P.R. China; ^2^ Department of Hematology, Fujian Institute of Hematology, Fujian Provincial Key Laboratory on Hematology, Fujian Medical University Union Hospital, Fuzhou 350001, P.R. China; ^3^ Zhangzhou Affiliated Hospital of Fujian Medical University, Zhangzhou 363000, P.R. China

**Keywords:** acute myeloid leukemia partially differentiated (FAB-M2), FAMLF-1 gene, miR-181a1, single nucleotide polymorphism haplotype, pathogenesis

## Abstract

Genetic heterogeneity is the basis of clinical heterogeneity among different subtypes of AML. We have successfully cloned a gene related to AML termed *FAMLF* from a FAB-M2 patient's sample of a second largest AML pedigree. Then we revealed at least three splice variants, named as *FAMLF-1*, *FAMLF-2* and *FAMLF-3*, and found miR181a1/b1 in the second intron of FAMLF gene family. Higher expression of FAMLF-1 was related to a higher complete remission (CR) rate, but shorter relapse free survival (RFS) in AML. We further found that the *FAMLF-1* single nucleotide polymorphism (SNP) haplotype and its expression were positively correlated to clinical parameters of acute myeloid leukemia partially differentiated (FAB-M2) patients, but not FAB non-M2 patients or Acute Monocytic Leukemia (FAB-M5) patients. GTAGG SNP haplotype of *FAMLF* gene might increase FAB-M2 susceptibility in Han population and act as a useful candidate biomarker for FAB-M2 screening. We also demonstrated that *FAMLF-1* gene silencing in FAB-M2 cells could lead to proliferation inhibition, cell cycle G0/G1 phase arrest, and differentiation promotion independent of its intronic miR-181a1, which might be related to Akt/c-Myc pathway. These findings reveal a role of *FAMLF-1* as a potential pathogenic gene for FAB-M2.

## INTRODUCTION

Acute myeloid leukemia (AML) is a clinically and genetically highly heterogeneous disease that accounts for 65.7% of acute leukemia [1]. Over the past decades, the prognosis of AML has improved, approximately 60–70% of AML patients experience long-term survival [2]. Acute Promyelocytic Leukemia (APL) is now characterized by CR rates of 90% and cure rates of about 80%, which is now considered to be the most curable subtype of AML [3]. FAB-M2 and FAB-M5 are both the most common morphologically classified subtypes of AML, moreover the former has better OS than the latter among every age groups [1]. However, long-term OS of non-M3(including FAB-M2 and FAB-M5) subtypes is still poor. Genetic heterogeneity including gene mutation and expression abnormity is the basis of these clinical heterogeneity among different subtypes of AML [4, 5]. Therefore, discovering and studying novel AML related genes may help to illustrate the leukemogenesis mechanism, especially to improve treatment outcomes of FAB non-M3.

**Figure 1:**
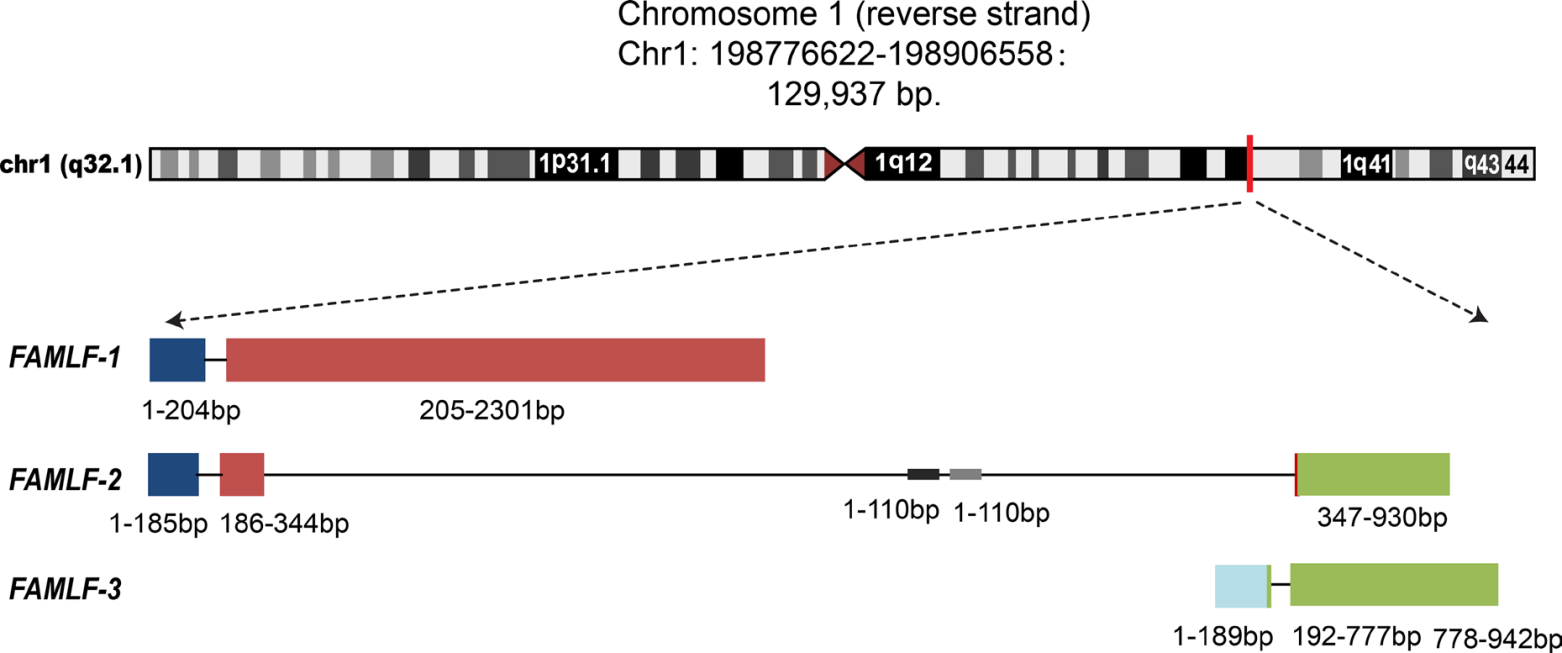
Three alternative splicing isoforms of FAMLF gene family, FAMLF-1 (2313 bp), FAMLF-2 (930 bp), FAMLF-3 (942 bp), and it’ intronic miRNA embedded in human chromosome 1q32.1 genomic Note: 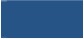 represents exon1, 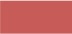 represents exon 2, 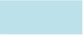 represents exon 3, 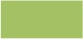 represents exon 4. Different transcripts of FAMLF gene family shared exon 1, 2 or 4. 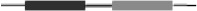 represents the genomic sequence of miR-181a1 and miR-181b1, embedded at the area of the second intron in FAMLF gene sequences. The construction of three splice variants of *FAMLF* are following: *FAMLF-1(FAMLF)* = *Exon1* + *Exon2*, *FAMLF-2* (*MIR181A1HG*, lncRNA)= *Exon1* + *Exon2’* + *Exon4’, FAMLF3*(*MIR181A1HG*, lncRNA) = *Exon3*+*Exon4*.

Familial acute myeloid leukemia has unique research value to find AML related genes, which can help to elucidate the leukemogenesis and improve the outcome of AML [6–10]. During the previous study, we had successfully cloned a gene, called Homo sapiens familial acute myelogenous leukemia related factor (*FAMLF*), from a FAB-M2 patient's bone marrow sample of a large AML pedigree with 11 cases in four consecutive generations, which was the second largest AML pedigree around the world [11, 12]. In this pedigree, most of members (6/11) were FAB-M2 patients. Using bioinformatics, we revealed the existence of at least three splice variants of *FAMLF*, named as *FAMLF-1*, *FAMLF-2* and *FAMLF-3*, and also found miR181a1/b1 embedded in the second intronic regions of *FAMLF* gene family (see Figure 1). Furthermore, we were able to detect a doublet at 8 kDa by Western blot analysis of total protein lysate from AML patient's cell and AML cell lines [13]. The expression of *FAMLF-1* was significantly higher in de novo adult AML than the control. Higher expression of *FAMLF-1* was related to a higher CR rate, but shorter RFS in AML [14]. So we speculate *FAMLF-1* may play a role in the development of AML, just like BCR/ABL fuse gene's role in Ph+-ALL [15].

To confirm the clinical significance of *FAMLF-1* expression in different subtypes of AML, we further used similar statistical methods to analyze the relationship between the *FAMLF-1* expression and its clinical hematology characteristics in the same 132 de novo AML cohort reported as before [14]. We further found correlation between *FAMLF-1* expression and clinical parameters of FAB-M2 patients, but not FAB non-M2 patients or FAB-M5 patients (Details are described in the discussion, also shown in Supplementary Table 2). Therefore, we further speculate *FAMLF-1* may play a role in the development of FAB-M2, but not in FAB-M5.

So far, the mutation spectrum of *FAMLF-1* has not been reported in different subtypes of sporadic AML, furthermore *FAMLF-1*'s function has not been reported. In the present study, we investigated the clinical significance of *FAMLF-1* mutations in a large cohort of AML patients including the second largest AML family members, and studied the function of *FAMLF-1* in FAB-M2 cells through lentiviral mediated gene knockdown.

## RESULTS

### Genomic variations spectrum of *FAMLF* gene family in the AML pedigree members

**Figure 2:**
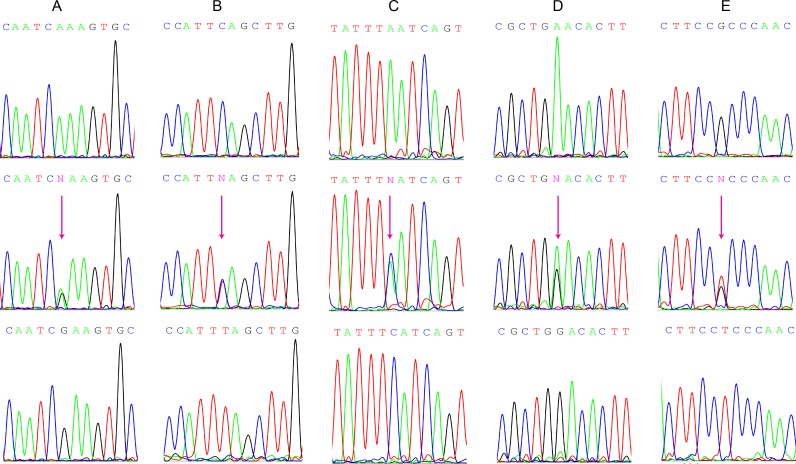
Genomic DNA Sanger sequencing of the 5 different SNP genotypes in FAMLF gene family (**A**) SNP1 contained three genotypes: AA、AG、GG. (**B**) SNP2 contained three genotypes: CC、CT、TT. (**C**) SNP3 contained three genotypes: CC、CA、AA. (**D**) SNP4 contained three genotypes: GG、GA、AA. (**E**) SNP5 contained three genotypes: GG、GT、TT.

The genotypes of *FAMLF* gene were confirmed by Sanger sequencing. We found previously unreported 5 SNPs in *FAMLF* gene family (Figure 2), and submitted to NCBI ClinVar SNP database acquired the accession number: SCV000577909, SCV000577910, SCV000577911, SCV000577912, SCV000577913, and SCV000577913, shorting for SNP1, SNP2, SNP3, SNP4, and SNP5 correspondingly.The genotypes of these SNPs in the reverse strand of human genome are described as following: SNP1 contained three genotypes; AA,AG and GG; SNP2 contained three genotypes; CC, CT and TT; SNP3 contained three genotypes; CC, CA and AA; SNP4 contained three genotypes; GG, GA and AA; SNP5 contained three genotypes; GG, GT and TT.

The SNP genotypes and SNP alleles distributions of *FAMLF* gene family in 8 cases of pedigree members and 65 cases of controls were described in Online Supplementary Table 6. The genotype distributions of each two examined polymorphisms complied with Hardy-Weinberg equilibrium in both patients and controls (*P* > 0.05).

There were no significant differences in the allele, genotype of SNP4 and SNP5 (*P* > 0.05). The alleles frequency of SNP1, SNP2 and SNP3 were significant different between AML family members and healthy controls (*P* = 0.0004, *P* = 0.0029, *P* = 0.0029, respectively). The genotype of SNP1, SNP2 and SNP3 were significant different between AML family members and healthy controls (*P* = 0.0009, *P* = 0.0037, and *P* = 0.0037). The frequency of ACCAT haplotype in *FAMLF* gene family was significantly higher in AML pedigree members with AML or high risk of the disease (45.6%) than in controls (5.35%) In other words, ACCAT haplotype carriers had a 1414.7% increased risk of AML relative to healthy controls in this AML pedigree (95% CI: 4.771–41.954; *P* < 0.0001) .

### Genomic variations spectrum of *FAMLF* gene family and its clinical significance in sporadic AML

### Characteristics of the Study Population

The clinical characteristics of the study population were summarized in Online Supplementary Table 7. There were no significant deviations in the distribution of age and gender (*P* = 0.227 and 0.156) between patients and controls.

### The relationship between the *FAMLF* genotype and different FAB AML subtype

There were also the same 5 SNPs in *FAMLF* gene family in sporadic AML patients with different frequencies as the pedigree members. The SNP genotypes and SNP allele distributions of *FAMLF* gene family in the 93 cases of AML patients, 60 cases of FAB non-M2 patients, 41 cases of FAB-M5 patients and 65 cases of healthy controls are described in Online Supplementary Tables 8 and 9. The genotype distributions of each two examined polymorphisms complied with Hardy-Weinberg equilibrium in both patients and controls (*P* > 0.05). There were no significant differences in the allele, genotype and haplotype distributions of 5 SNPs between total AML patients, FAB non-M2 patients, FAB-M5 patients and healthy controls (*P* > 0.05).

The SNP genotypes and SNP allele distributions of *FAMLF* gene family in 33 cases of FAB-M2 patients and 65 cases of controls were described in Online Supplementary Table 8. The SNP genotype distributions of each two examined polymorphisms complied with Hardy-Weinberg equilibrium in both patients and controls (*P* > 0.05). There were no significant differences in the allele, genotype of SNP1, SNP2 and SNP3 (*P* > 0.05). The genotype of SNP4 and SNP5 were significant different between FAB-M2 patients and healthy controls (*P* < 0.001, and *P* = 0.0116). The frequency of GTAGG haplotype of *FAMLF* gene family was significantly higher in patients (40.91%) than in controls (25.53%). In other words, GTAGG haplotype carriers had a 192.3% increased risk of AML relative to controls (95% CI: 1.0201–3.6250; *P* = 0.0419) .

### The expression levels of *FAMLF-1* in FAB-M2 patients with difference GTAGG haplotype

**Figure 3:**
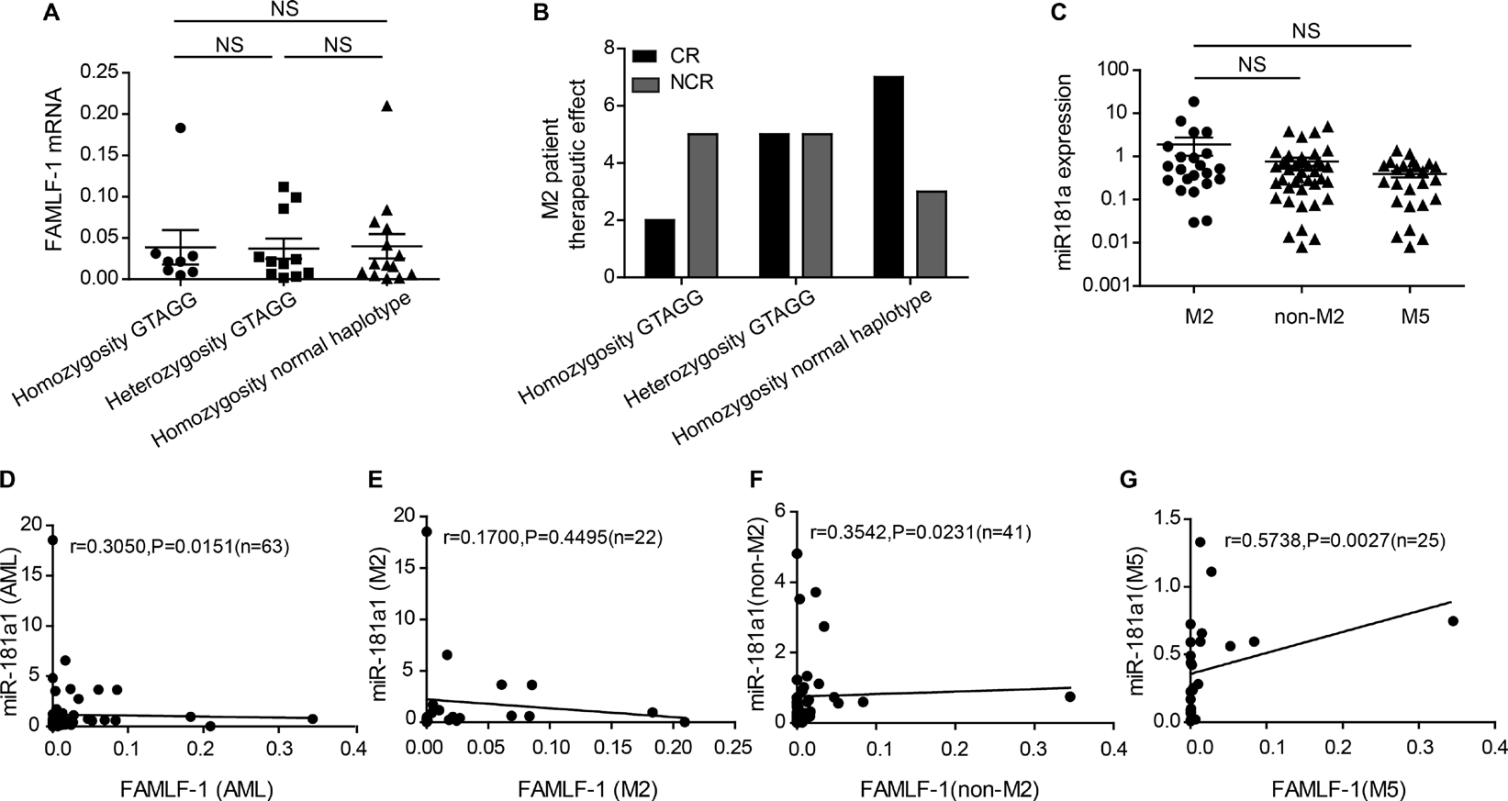
Clinical significance of haplotype GTAGG in FAB-M2, relationship between the expression level of miR-181a1 and FAMLF-1 in AML (**A**) Comparation the expression of *FAMLF-1* in FAB-M2 among disease haplotypes and normal haplotype(*P* > 0.05). (**B**) Comparation the CR rate in FAB-M2 among disease haplotypes and normal haplotype(*P* > 0.05). (**C**) The expression level of miR-181a1 in patients with FAB-M2, FAB non-M2 or FAB-M5(*P* > 0.05). (**D**) The relationship between the expression level of miR-181a1 and *FAMLF-1* in patients with AML (*P* = 0.0151). (**E**) The relationship between the expression level of miR-181a1 and *FAMLF-1* in patients with FAB-M2 (*P* > 0.05). (**F**) The relationship between the expression level of miR-181a1 and *FAMLF-1* in patients with FAB non-M2 (*P* = 0.0231). (**G**) The relationship between the expression level of miR-181a1 and *FAMLF-1* in patients with FAB-M5 (*P* = 0.0027).

As shown in Figure 3A, the expression levels of *FAMLF-1* was not significant different among homozygous GTAGG, heterozygosity GTAGG and normal haplotype in FAB-M2 patients. It was indicated that the pathogenic haplotype of GTAGG did not affect the expression of *FAMLF-1*, so the pathogenic haplotype of *FAMLF* gene family might participate in the development of FAB-M2 through other genetic pathways.

By Kruskal-Wallis test, we did not find a significant correlation between the alleles or genotypes of SNP1, SNP2, SNP3, SNP4, SNP5 and the expression level of *FAMLF-1* or other clinical hematology features in FAB-M2 patients.

### Comparison of CR rate between difference GTAGG haplotypes in patients with FAB-M2

As shown in Figure 3B, from homozygous GTGG (CR rate of 28.6%, *n* = 7), heterozygous GTGG (CR rate of 50%, *n* = 10) to homozygous wild type (CR rate of 70% *n* = 10) in FAB-M2 patients, the effect of chemotherapy-induced CR rate improved. However, due to small number of patients among the groups, there were no significant differences between the three groups using the chi-square test (*P* > 0.05). Also, because of the small number cases, we failed to carry out OS and RFS analysis in patient with FAB-M2.

### A positive correlation between *FAMLF* and miR-181a expression in AML and FAB-M5 patients, but not in FAB-M2

We subsequently examined the miR-181a expression in PBMCs of patients with AML (*n* = 63) and healthy controls (*n* = 20). The expression of miR-181a1 was not significant different among FAB-M2, FAB non-M2 and FAB-M5 patients (*P* > 0.05) (Figure 3C). A statistically significant correlation was found between *FAMLF-1* and miR-181a expression levels in patients with AML (*R* = 0.3050; *P* = 0.0151), FAB non-M2 (*R* = 0.3542; *P* = 0.0231), FAB-M5(*R* = 0.05738; *P* = 0.0027), but not in patients with FAB-M2(*R* = 0.1700; *P* = 0.4495). Figure 3D–3G are scatter plots of the *FAMLF-1* expression versus the miR-181a expression showing the correlation between the two parameters in 4 different groups of AML patients.

### Knockdown of *FAMLF-1* significantly decreased cell growth, inhibited cell cycle, and increased differentiation of Kasumi-1 cells *in vitro*

In this study we designed four siRNA sequences to construct the corresponding recombinant lentiviral vector transducted into U937 and Kasumi-1 cells(Four targets of siRNA shown in Online Supplementary Table 5). According to the results of the knockdown efficiency, we selected siRNA 1548 as an RNAi target sequence, and ultimately constructed *FAMLF-1* RNAi vector. The sequence of recombinant plasmid was verified by Sanger sequencing and BLAST alignment analysis: the inserted DNA fragment sequences was correct, showing that GV248-GFP-*FAMLF-1*-shRNA recombinant vector was constructed successfully. And *FAMLF-1*-RNAi-LV virus titers reached 6 ×10^8^ TU/ml.

### *FAMLF-1*-RNAi-LV knockdown FAMLF-1 in U937 and Kasumi-1 cells

By real-time quantitative RT-PCR (qRT-PCR) analysis we found that the expression of *FAMLF-1* was significantly inhibited in U937 and Kasumi-1 cells after *FAMLF-1*(1548)-RNAi-LV infection (Online Supplementary Table 10). The inhibition rate of *FAMLF-1* was 83% in Kasumi-1 cells and 74% in U937 cells after *FAMLF-1*(1548)-RNAi-LV infection (*P* < 0.05).

After *FAMLF-1* gene silencing, cell growth and clone formation were inhibited, cell cycle was arrested in Kasumi-1 cells but not affected the cytobiology characteristic of U937 cells (data not shown).

### Cell proliferation assay of Kasumi-1 cells

**Figure 4:**
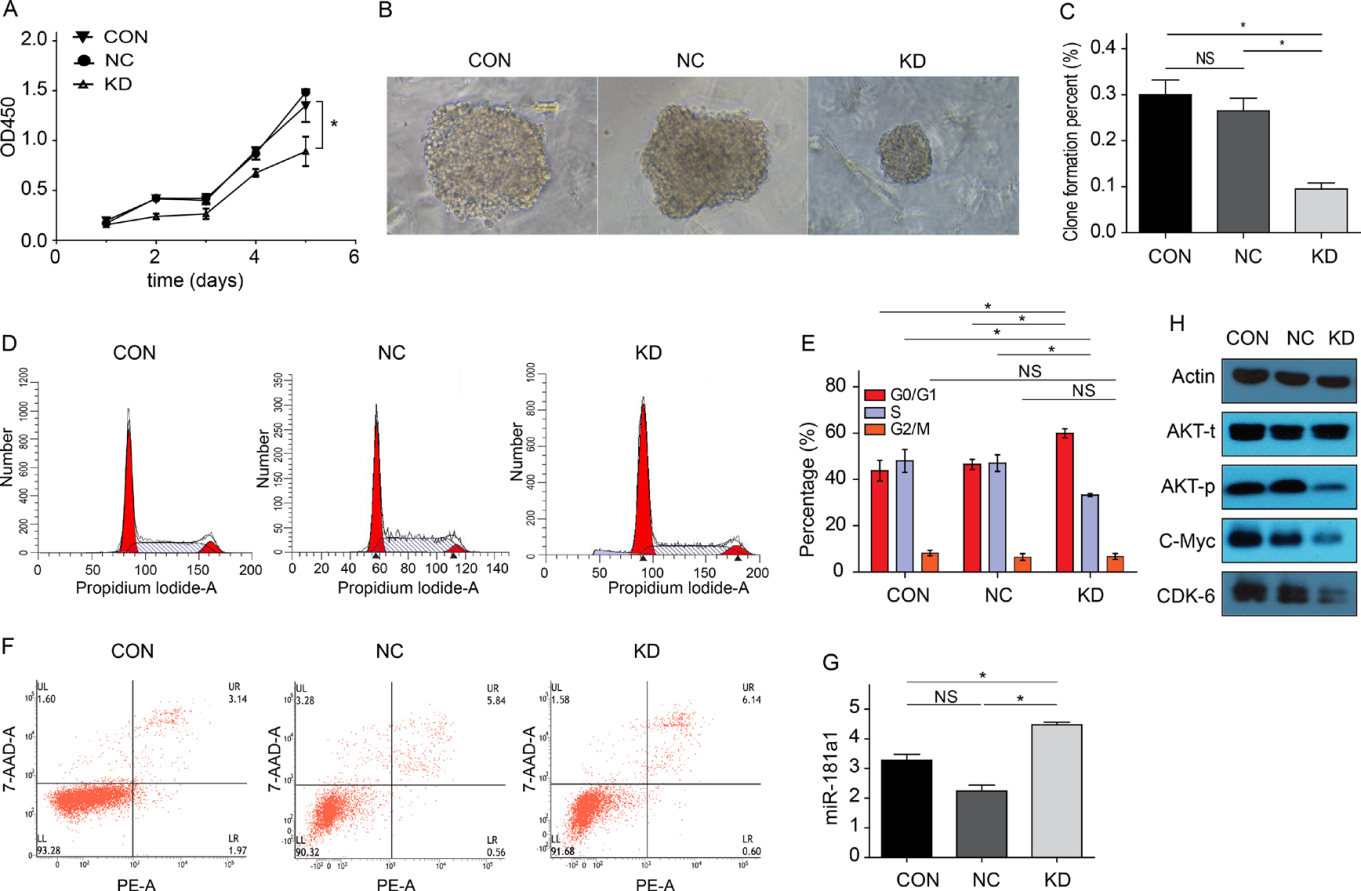
Cytobiology characteristics changes of Kasumi-1 cells after FAMLF-1 gene silencing (**A**) Proliferation changes of Kasumi-1 cells after *FAMLF-1* gene silencing(*P < 0.05). (**B**) Colony formation size change of Kasumi-1 cells after *FAMLF-1* gene silencing. (**C**) Colony formation number change of Kasumi-1 cells after *FAMLF-1* gene silencing(*P < 0.05). (**D**) FACS measuring the cell cycle change of Kasumi-1 cells after *FAMLF-1* gene silencing. (**E**) FACS measuring the cell cycle percent change of Kasumi-1 cells after *FAMLF-1* gene silencing(*P < 0.05). (**F**) Annexin V-PE/7AAD apoptosis assay of Kasumi-1 cells after *FAMLF-1* gene silencing. (**G**) MiR-181a expression levels changes of Kasumi-1 cells after *FAMLF-1* gene silencing(*P < 0.05). (**H**) AKT protein phosphorylation status, CDK6 and c-Myc protein expression changes of Kasumi-1 cells after *FAMLF-1* gene silencing.

As shown in Figure 4A, after *FAMLF-1* gene silencing, the proliferation of Kasumi-1 cells was inhibited in 24 h, 48 h,72 h, and 96 h of cell growth curve (*P* < 0.05), and inhibited most seriously at 96 hours.

### Clone formation assay of Kasumi-1 cells

As shown in Figure 4B and 4C, colony formation of Kasumi-1 cells were significantly inhibited after *FAMLF-1* gene silencing.

### Cell cycle detection

As shown in Figure 4D and 4E, after *FAMLF-1* gene silencing in Kasumi-1 cells, we found that the cells in G0/G1 phase increased and the cells in S phase reduced compared with the CON and NC groups. However, the cells in G2/M phase remained unchanged. Thus, it can lead to Kasumi-1 cells arrested in G0/G1 phase after *FAMLF-1* gene silencing.

### Flow cytometry apoptosis detection

Seen from Figure 4F, apoptosis rate was not significant different in Kasumi-1 cells after *FAMLF-1* gene silencing compared with the CON and NC groups, (*P* > 0.05).

### FCM detection expression levels of cell surface CD11b, CD13, CD33, CD34

**Figure 5:**
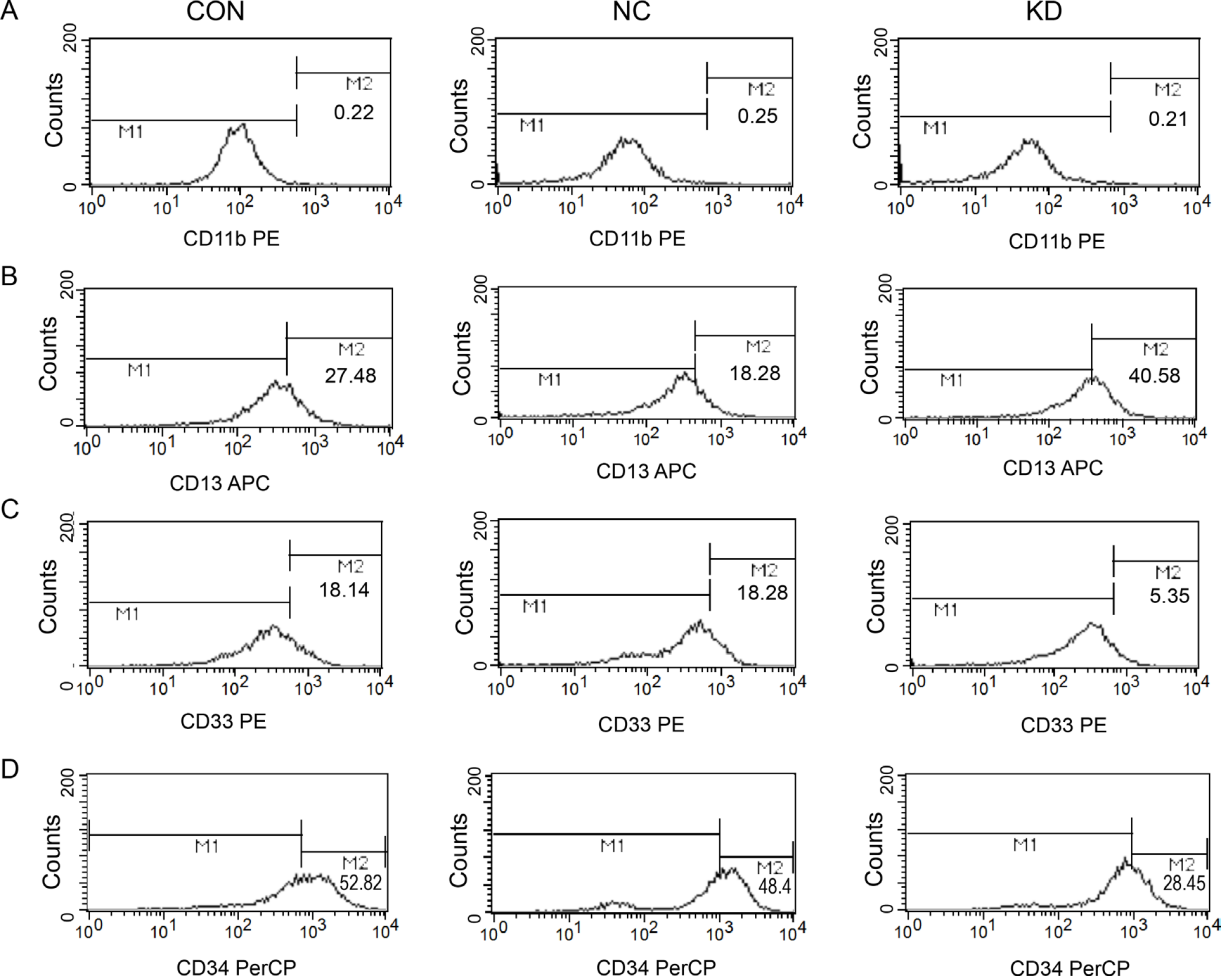
Effect of surface Cluster of differentiation expression levels changes in Kasumi-1 cells after *FAMLF-1* gene silencing (**A**) FACS detection surface expression of CD11b in Kasumi-1 cells after *FAMLF-1* gene silencing. (**B**) FACS detection surface expression of CD13 in Kasumi-1 cells after *FAMLF-1* gene silencing. (**C**) FACS detection surface expression of CD33 in Kasumi-1 cells after *FAMLF-1* gene silencing. (**D**) FACS detection surface expression of CD34 in Kasumi-1 cells after *FAMLF-1* gene silencing.

Seen from Figure 5A, after *FAMLF-1* gene silencing, CD11b expression level of Kasumi-1 cells was no significant difference from the CON and NC groups (*P* > 0.05). Seen from Figure 5B, after *FAMLF-1* gene silencing, CD 13 expression level of Kasumi-1 cells was significant higher than the CON and NC groups (*P* < 0.05). Seen from Figure 5C, after *FAMLF-1* gene silencing, CD33 expression level of Kasumi-1 cells was significant lower than the CON and NC groups (*P* < 0.05). Seen from Figure 5D, after *FAMLF-1* gene silencing, CD34 expression level of Kasumi-1 cells after *FAMLF-1* gene silencing was significant lower than the CON and NC groups (*P* < 0.05).

### Detection of miR-181a expression in Kasumi-1 cells

Seen from Figure 4G, after *FAMLF-1* gene silencing, we can find the expression level of miR181a1 in Kasumi-1 cells increased compared with the CON and NC groups.

Endogenous expression of the miR-181a1 was significantly upregulated by forced inhibition of *FAMLF-1 in vitro*.

### Proliferation and cell cycle related proteins detection

Seen from Figure 4H, after *FAMLF-1* gene silencing, phosphorylation state of AKT protein was inhibited, c-Myc and CDK6 proteins were downregulated inducing cell cycle G0/G1 phase arrested, resulting in cell proliferation inhibited in Kasumi-1 cells compared with the CON and NC groups.

Seen from Supplementary Figure 3, after *FAMLF-1* gene silencing, Cyclin D, Cyclin E, CDK2, P53 and Chk1/2 associated with cell cycle G1/S arrested and cell proliferation inhibition were no significant change compared with the CON and NC groups.

### Detection of *FAMLF-2* and *FAMLF-3* expression in Kasumi-1 cells

Seen from Supplementary Figure 2, we can find the expression level of *FAMLF-2* and *FAMLF-3* in Kasumi-1 cells were not change comparing with the CON and NC groups, after *FAMLF-1* gene silencing.

Endogenous expression of the *FAMLF-2* and *FAMLF-3* were not affected by the inhibition of *FAMLF-1 in vitro*.

## DISCUSSION

Many AML-related gene expression abnormalities and/or gene mutations are involved in leukemogenesis and development of AML. Familial acute myeloid leukemia has unique research value to find the genetic heterogeneities of AML. These genetic abnormalities are also helpful for individual treatment and prognosis evaluation [6–10]. In the present study, we investigated the clinical significance and prognostic value of *FAMLF-1* expression and *FAMLF* gene family mutations in the AML pedigree and a large cohort of de novo AML.

Our previous study found that the *FAMLF-1* expression in AML was correlated with hemoglobin level, HBDH level, and peripheral blood blasts percentage [14]. By Mann-Whitney *U* test, Kruskal-Wallis H test and chi-square test again, we reanalyzed the relationship between the expression of *FAMLF-1* and its clinical characteristics in the same AML cohort reported as before [14]. Interestingly, we further found correlation between the *FAMLF-1* expression and clinical parameters in FAB-M2, but not in FAB non-M2 and FAB-M5 (Online Supplementary Table 2). *FAMLF-1* expression in FAB-M2 was correlated with WBC count (*P* = 0.0019), hemoglobin level (*P* = 0.0011), percentage of peripheral blood blasts (*P* < 0.0001), HBDH level (P= 0.0025; Online Supplementary Table 2). The expression levels of *FAMLF-1* in FAB-M2 was higher than FAB non-M2, FAB-M5 (*P* = 0.0152, *P* = 0.0078, Online Supplementary Figure 1A). The higher expression of *FAMLF-1* had higher CR rate in FAB-M2 (P = 0.0407), but not in FAB-M2 and FAB-M5 (*P* > 0.05) (Online Supplementary Figure 1B). Thus, the expression of *FAMLF-1* was significantly associated with certain clinical characteristics of FAB-M2 but not FAB non-M2 and FAB-M5. *FAMLF-1* is likely to play an important role in the pathogenesis of FAB-M2, which is worth to further research mutation spectrum of *FAMLF* gene family and its clinical characteristics in FAB-M2.

Using Sanger sequencing, we found no mutations in *FAMLF-1*'s ORF region and miR181a1/b1 core functional sequence, suggesting that they were highly conserved sequence. This study did not find any mutations related to the expression levels of *FAMLF-1*, which suggests that expression of *FAMLF-1* may not be regulated by gene mutation. However, we detected 5 SNPs in *FAMLF* gene family with different frequencies in the AML pedigree members, healthy controls, and sporadic AML patients. Interestingly, the frequency of ACCAT haplotype of *FAMLF* gene family was significantly higher in AML pedigree members than in the controls. Our results showed that ACCAT haplotype carriers had a 1414.7% increased risk of AML compare to controls, suggesting *FAMLF* gene family is closely related to AML in the AML pedigree. The study showed that 5 SNPs corresponding ACCAT haplotype of *FAMLF* gene family was a risk haplotype in AML pedigree members, so ACCAT haplotype of *FAMLF* gene family can be used as a biomarker for screening the risk of AML in the AML pedigree. The genetic abnormalities associated with familial AML can be extended to the sporadic AML as reported [16]. Moreover, we found the frequency of GTAGG haplotype of *FAMLF* gene was significantly higher in FAB-M2 patients than in controls. It was shown that GTAGG haplotype carriers had a 192.3% increased risk of FAB-M2 compare to controls, suggesting *FAMLF* gene family is closely related to FAB-M2. The study firstly showed that GTAGG haplotype of *FAMLF* gene family was a risk haplotype in patients with FAB-M2 but not AML, FAB non-M2 or FAB-M5. GTAGG haplotype of *FAMLF* gene family can be used as a biomarker for screening the risk of FAB-M2 in the Han population. These studies significantly demonstrated a contribution of GTAGG haplotype of *FAMLF* gene family to FAB-M2 susceptibility in Han population. Although the number of cases in this article is still limited, it provides a non-damaging mean or possible direct evidence of laboratory screening for FAB-M2. Moreover, it also established background data for further studying the mechanisms of *FAMLF-1* gene in the leukemogenesis and development of FAB-M2.

Both the expression profile and mutation spectrum of *FAMLF-1* were closely related to FAB-M2 but not FAB non-M2 and FAB-M5, which consistently suggested that *FAMLF-1* might be closely related to leukemogenesis or development of FAB-M2. However, how *FAMLF-1* involves in FAB-M2 cell remains speculative. Hence, we further investigated the specific role of *FAMLF-1* in Kasumi-1 cells established from FAB-M2 patient with abnormally over-expression of *FAMLF-1* by lentivirus mediated RNA interference.

After *FAMLF-1* gene knockdown in Kasumi-1 cells, it could lead to Kasumi-1 cell cycle G0/G1 arrested, cell proliferation and colony formation inhibited, compared with the CON and NC groups. Moreover, after *FAMLF-1* gene knockdown, CD13 expression levels of Kasumi-1 cell line was increased, while CD33 and CD34 expression levels were significantly reduced, suggesting the *FAMLF-1* gene may be involved in the regulation of Kasumi-1 cell differentiation, which are in according with the clinical significance of *FAMLF-1* expression profile analysis results [14]. As reported that the most important biological characteristics of AML cells are proliferation over-activated, cell cycle disorganized, differentiation blocked, and apoptosis inhibited [17]. We can reasonably speculate *FAMLF-1* involves in FAB-M2 through regulation of cell cycle, proliferation, and differentiation. In order to illustrate the underlying molecular mechanisms of *FAMLF-1* involvement in FAB-M2, we detected the expression of the signaling pathway proteins related to cell cycle G1/S checkpoint regulation by western blotting. As a result, we found the *FAMLF-1* gene silencing in Kasumi-1 cells could lead to inhibition of Akt phosphorylation status, down-regulation of c-Myc protein and inhibition of CDK6 activity (Figure 4 H).

Akt signaling pathway plays a key role in cell cycle regulation and cell proliferation process [18]. Akt can activate CDK6 [19] and promote cells to pass G1 phase checkpoint. Meanwhile, activation of AKT signaling pathway is one of the main mechanisms to regulate the expression of c-Myc [20]. C-Myc can function as transcription factors, playing an important role in a variety of cell behavior such as cell proliferation and differentiation, which directly regulate the expression of CDK4/6 and Cyclin D, involving in the regulation of cell cycle regulation and cell proliferation [21]. Moreover CDK6 is a cyclin-dependent kinase, combining with cyclins related protein and CDK inhibitors to form the corresponding complexes, to regulate G1/S phase transition, promoting the cells to pass the G1 phase checkpoint. Therefore, after Akt pathway inhibition, it may consequently decrease the activity of CDK6 which leads to cell cycle arrest in G1 phase. Moreover, Akt inhibition could also leads to downregulation of c-Myc. Both of them can inhibit the expression of CDK6, leading to cell cycle arrest in G1 phase, and cell proliferation inhibition [22]. Downregulation of c-Myc may also inhibit cell differentiation in normal and leukemic human myeloid progenitor cells [23]. Moreover, we also found that Cyclin D, Cyclin E, CDK2, P53 and Chk1/2 associated with cell cycle G1/S arrested and cell proliferation inhibition were no significant change after *FAMLF-1* gene silencing (Supplementary Figure 3). Combined with biological characteristic changes after knockdown of *FAMLF-1* in Kasumi-1 cells mentioned above, we can reasonably speculate *FAMLF-1* gene silencing can result in Kasumi-1 cells cycle G0/G1 phase arrested, cell proliferation inhibited and cell differentiation promoted through Akt phosphorylation inhibited, c-Myc down-regulated and CDK6 activity inhibited, which is similar to Akt signal pathway findings the other scholars mentioned above.

MiR-181a1 is embedded in the second intron of *FAMLF* gene (Figure 1), so *FAMLF-1* is the host gene of miR-181a1. Typically host genes co-express with intronic miRNA, both of which synergistically play a similar biological function [24]. More importantly, we found a positive correlation between *FAMLF-1* and its miR-181a expression in patients with AML, FAB non-M2, FAB-M5, but not in patients with FAB-M2. Studies have demonstrated that the miRNA-181 family plays an important role in the development of AML [25, 26]. Schwind S et al. [27] reported that higher expression of miR-181a in patients with CN-AML was significantly associated with higher hemoglobin and percentage of circulating blasts, FAB-M1 and FAB-M2 subtypes. Moreover, patients with higher miR-181a expression had a higher CR rate, as well as a longer DFS and OS. Our study showed that the *FAMLF-1* expression of patients with FAB-M2 was positively correlated with the percentage of peripheral blood blasts and that higher *FAMLF-1* expression in FAB-M2 was associated with a higher CR rate. Our results of *FAMLF-1* mentioned above are similar to miR-181a1 findings to some extent [27]. However, the expression of intronic miR-181a was not correlated with the host gene *FAMLF-1* in patients with FAB-M2. So we presume that *FAMLF-1* participate in the development of FAB-M2 independent of its intronic miR-181a1.

After *FAMLF-1* gene knockdown in Kasumi-1 cells, the expression levels of intronic miR181a1 did not decrease, it may be further inferred that *FAMLF-1* gene silencing of Kasumi-1 cells result in proliferation inhibited and cell cycle G0/G1 phase arrested independent on down-regulation of its intronic miR-181a1.

*FAMLF-1*, *FAMLF-2* and *FAMLF-3* are the three kinds of splice variant transcriptions belong to *FAMLF* gene family. None of their biology function has been reported before. In this study, we found that the expression levels of *FAMLF-2* and *FAMLF-3* did not changed after *FAMLF-1* gene knockdown in Kasumi-1 cells (Supplementary Figure 2), it may be further inferred that *FAMLF-1* gene silencing of Kasumi-1 cells result in proliferation inhibited and cell cycle G0/G1 phase arrested which was not dependent on *FAMLF-2* and *FAMLF-3*. Thus, the biological characteristic changes after *FAMLF-1* gene silencing are independent of *FAMLF-2* and *FAMLF-3*. Additionally, lentivirus mediated RNA interference targeting *FAMLF-1* in U937 cells which have been established from acute monocytic leukemia(FAB-M5) did not result in significant biological characteristic changes (data no shown). Neither the expression of *FAMLF-1* nor the genomic variations spectrum of *FAMLF* gene family was associated with FAB-M5 patients. Therefore, we concluded that *FAMLF-1* may not function in the leukemogenesis of FAB-M5, suggesting that gene expression and variation analysis can be used as an important basis for the prediction of genes’ function in the disease and its subtypes.

In summary, our study showed that ACCAT haplotype of *FAMLF* gene family was a risk haplotype in the AML pedigree members with AML or high risk of AML, and GTAGG haplotype of *FAMLF* gene family was a risk haplotype in patients with FAB-M2. *FAMLF-1* was over-expressed in immature hematopoietic cells (such as FAB-M2 ), and its expression was significantly related to WBC count, percentage of peripheral blood blasts, hemoglobin level, and HBDH level in the FAB-M2, but not FAB non-M2 and FAB-M5. *FAMLF-1* over-expression in FAB-M2 patients correlates with favorable CR rate but not the long-term outcome. There was a positive correlation between *FAMLF-1* and miR-181a expression in patients with AML, FAB non-M2, FAB-M5, but not in patients with FAB-M2. *FAMLF-1* is a gene with complex functions independent on its intronic miR-181a1 in FAB-M2, which can participate in the regulation of cell cycle, cell proliferation and cell differentiation processes, which might play a role in the development of FAB-M2. Further studies are needed to elucidate the role of *FAMLF-1* in the leukemogenesis of FAB-M2 and the underlying mechanisms. Overall, our current study may provide important insight for uncovering the role of *FAMLF-1* in regulating FAB-M2 cell proliferation and differentiation.

## MATERIALS AND METHODS

### Patient samples

The pedigree with 40 members, including 11 AML patients in four consecutive generations, was closely followed for over three decade [12], and blood samples were obtained from 21 family members. A thorough clinical evaluation, including morphological, cytochemical and cytogenetic analysis of peripheral blood and/or bone marrow, was performed in affected members. The 13 family members [12] were recruited as patients or high risk of AML (Shown in Supplementary Table 1). 11 out of the 13 family members have been reported of containing a high risk of AML [12], and were used for DNA sequence analysis of the *FAMLF* gene family.

Other subjects included a total of 158 participants of Han Chinese descent who were recruited from Fujian Medical University Union Hospital from September 2012 to March 2015. The novel diagnosis of AML and its subtypes was determined according to FAB and WHO classifications system [18, 19]. Bone marrow aspiration, blood counts, flow cytometry, molecular study and cytogenetic analysis were performed, reviewed and classified accordingly. Patients were treated with standard procedures approved by the Fujian Institute of Hematology based on the 2008 NCCN guideline for AML [14]. After two courses of induction chemotherapy, achievement of CR and clinical outcomes of AML patients were assessed. CR was defined according to Cheson's criteria [17]. OS was calculated from the date of diagnosis until the date of death, censoring for patients alive at last follow-up. RFS was calculated from the date of the first CR until the date of the first relapse [17]. Sample processing was described in the online supplementary information. The de novo adult AML group involved 93 patients selected at random. Clinical information of patients, including age, sex and pathological parameters were also collected. Unaffected individuals (*n* = 65) of matched geographic ancestry were included as healthy controls. Genomic DNA was extracted from the peripheral blood of all participants using standard procedures according to the instruction. All experimental protocols were approved by the Ethics Committee of Fujian Medical University, and were conducted according to local laws and regulations and in accordance with the Declaration of Helsinki Principles.

### Statistics

All data were analyzed with SPSS version 18.0 software (SPSS Inc., Chicago, IL, USA). Continuous variables were expressed as median (min-max). Comparisons were made using unpaired *t*-test or nonparametric Mann-Whitney U test and Kruskal-Wallis test for continuous variables and *χ*^2^ test for categorical variables between groups. Correlations between continuous variables were calculated using the Spearman correlation coefficient.

Patients with rare recurrent cytogenetic aberrations such as t(7;12)(q36;p16), t(6;9)(p23;q34), t(16;21)(p11;q22), t(8;16)(p11;p13), monosomy 7, trisomy 8, and complex karyotype were classified as 1 cytogenetic subgroup entitled “rare cytogenetics.”

### Others molecular and cell biological methods

‘Cytogenetic and molecular analysis’, ‘DNA Extraction and Genotype analysis’, ‘Real-time quantitative PCR’, ‘Cell culture’, ‘Lentivector construction, packaging, and cell infection’, ‘Cell proliferation assay’, ‘Colony formation assay’, ‘Cell Cycle Analysis’, ‘Assessment of Apoptosis’ ,‘Differentiation determination’, and ‘Western blot’, are described in Supplementary materials and methods.

## SUPPLEMENTARY MATERIALS FIGURES AND TABLES







## Abbreviations

AML: Acute Myeloid Leukemia
APL: Acute Promyelocytic Leukemia
BLAST: Basic Local Alignment Search Tool
CD11b: Cluster of differentiation 11b
CD13: Cluster of differentiation 13
CD33: Cluster of differentiation 33
CD34: Cluster of differentiation 34
CR: Complete Remission
CT: Cycle Threshold
FAB: French–American–British classification systems
FAB-M2: Acute Myeloid Leukemia Partially Differentiated
FAB-M5: Acute Monocytic Leukemia
FAB non-M2: Non Acute Myeloid Leukemia Partially Differentiated
*FAMLF*: *Homo Sapiens Familial Acute Myelogenous Leukemia Related Factor*
*FAMLF-1*: *FAMLF splice variant 1*
*FAMLF-2*: *FAMLF splice variant 2*
*FAMLF-3*: *FAMLF splice variant 3*
GFP: Green Fluorescent Protein
HBDH: Hydroxybutyrate dehydrogenase
lncRNA: long non-coding RNA
LV: Lentiviral
miR-181a1/b1: microRNA-181a1/b1
mRNA: Messenger RNA
OD: Optical Density
ORF: Open Reading Frame
OS: Overall Survival
PBMCs: Peripheral Blood Mononuclear Cells
qRT-PCR: quantitative Reverse transcription-polymerase chain reaction
RFS: Relapse Free Survival
RNAi: RNA interference
shRNA: short hairpin RNA
siRNA: small interference RNA
SNP: single nucleotide polymorphisms
WHO: World Health Organization
