# Supplementary material for: Lentivirus-mediated RNA interference targeting FAMLF-1 inhibits cell growth and enhances cell differentiation of acute myeloid leukemia partially differentiated cells via inhibition of AKT and c-MYC
Source: Oncotarget. 2017 Sep 26;8(60):101372–82. doi: 10.18632/oncotarget.21276 (PMC5731881; doi:10.18632/oncotarget.21276)
Supplement: Supplementary file 1 [file oncotarget-08-101372-s001.pdf]

Lentivirus-mediated RNA interference targeting *FAMLF-1* inhibits cell growth and enhances cell differentiation of acute myeloid leukemia partially differentiated cells via inhibition of AKT and c-MYC

SUPPLEMENTARY MATERIALS

Sample processing

The healthy controls enrolled in this study were from the Medical Center of the Fujian Medical University Union Hospital. All participants in the study gave informed consent to use their specimens and access to clinical information for research, in accordance with the 2008 revised Declaration of Helsinki and the guidelines of the medical ethics committees of the Fujian Medical University Union Hospital.

The samples collected from the AML patients were analyzed at diagnosis. Peripheral blood was obtained from AML patients and normal individuals. Peripheral blood mononuclear cells (PBMCs) and bone marrow mononuclear cells (BMMCs) were isolated by Ficoll-Hypaque density gradient centrifugation.

Cytogenetic and molecular analysis

Leukemic samples were routinely investigated for cytogenetic aberrations using standard chromosome banding analysis by the national study groups. Leukemic samples were further analyzed for recurrent nonrandom genetic aberrations characteristic of AML as described by the World Health Organization (2008) classification of myeloid neoplasms and acute leukemia[17], including t(15;17)(q21;q22), inv(16)(p13q22), t(8;21)(q22;q22), t(9;22)(q34;q11), t(12;21)(p13;q22), and t(1;19)(q23;p13), using FISH and/or RT-PCR.

Detection of molecular aberrations included mutation analysis of, nucleophosmin 1 (NMP1), CEBPA, FMS-related tyrosine kinase 3 internal tandem duplication (FLT3-ITD), and C-KIT, this was confirmed by a multiplex ligation–dependent probe amplification analysis.

DNA extraction and Genotype analysis

Two milliliters of venous or bone marrow blood was taken from each patient before chemotherapy and healthy controls. Genomic DNA was extracted from PBMCs or BMMCs using TIANamp Blood DNA Kit (Tiangen Biotect, China). *FAMLF-1* genotypes were detected using Sanger gene sequencing analysis technique.

Briefly, PCR primers were first designed to detect the sequence of *FAMLF* gene family's 4 exons, cord sequence of miR 181a1/b1 and their associated splice sites using primer design software(Online Supplementary Table 3). The experimental conditions were shown in Online Supplementary Table 4 and amplification parameters were 94°C for 5 minutes, 30 cycles of 98°C for 10 sec, 56°C for 15 sec, 72°C for 30 sec with a final extension step at 72°C for 7 minutes. The PCR products were purified for sequencing after electrophoresis on an agarose gel. For sequencing, a Perkin Elmer Big Dye Sequencing kit (Perkin-Elmer, Shelton, CT, USA) and an ABI PRISM7700 sequencer (Perkin-Elmer ABI, Foster City, CA, USA) were used. The sequence datas were analyzed using Variant Reporter (Applied Biosystems).

RNA extraction, reverse transcription and real-time quantitative PCR

Procedures for RNA extraction and reverse transcription were performed according to the manufacturer's instructions. Briefly, total RNA was extracted from PBMCs by using a Trizol reagent (Invitrogen, Carlsbad, CA, USA) according to our established protocols [14]. Two micrograms of total RNA were used for cDNA synthesis using a cDNA reverse transcriptase kit (Thermo, Foster City, CA, USA) in accordance to the conditions described previously [14]. OligT18 primers were used for mRNA RT, and specific primers(TaqMan(R) MicroRNA RT KIT, Taqman, Life, number 4366596) were pre-designed for the miRNA RT reactions. The RT reaction mixture contained 0.15 ul of dNTPs (100 mM ,with dTTP), 1.0 ul of MultiScribe™Rever Transcriptase(50U/uL), 1.5 ul of Reverse Transcription Buffe (10×), 0.19 ul of RNase Inhibitor (20 U/uL), 4.16 ul of Nuclease-free water, 5 ul of total RNA, and 3ul RT primer. Mixing (5×). The 15 uL RT reactions were incubated in a 96-well plate for 30 minutes at 16°C, 30 minutes at 42°C, 5 minutes at 85°C. The reaction product can be saved from –15 to –20°C in short term.

For quantification of *FAMLF-1* expression reported as before[14], we used a forward primer (5′-TGTGCTCTATGGGTGTCAGG-3′), reverse primer (5′- CATTTGTTGGTGATCCTGCT-3′). For quantification of *FAMLF-2* expression, we used a forward primer (5′-ACCGTTTTGAAATTAGATCC-3′), reverse

primer (5′-GCAGAGCGAGACCTTAATC-3′). For quantification of *FAMLF-3* expression, we used a forward primer (5′-CAATCAACCCATCAAACG-3′), reverse primer (5′-GGAAGCCAAATTCACCC-3′). QRT-PCR was performed using a real-time PCR System with a SYBR master mix (Roche, Indianapolis, IN, USA). Quantitative PCR reactions contained 1 uL of cDNA, 0.3 uL of forward primer (10 pmol/uL), 0.3 uL of reverse primer (10 pmol/uL), 12.5 uL of FastStart Universal SYBR Green Master Mix (ROX), and 10.9ul of nuclease-free water. The 25 uL reactions were incubated in a 96-well plate for 2 minutes at 50°C, 10 minutes at 95°C, followed by 40 cycles of 15 seconds at 95°C, and 1 minute at 55°C. Quantitative PCR reactions were performed on the ABI/PRISM 7500 Sequence Detection system. Samples were considered eligible for testing only when the threshold cycle value (Ct) of the reference gene beta-actin was lower than 26.

For quantification of miR181a1 reported as before[14], a TaqMan microRNA Assay (Taqman, number4427975) was used. The reaction mixture contained 1.0 ul of Taqman® Small RNA Assay (20×), 1.33 ul of RT reaction production, 10.0ul of Taqman® Universal PCR Master Mix II (2×, no UNG), 7.67ul of Nuclease-free water. The 20 uL RT reactions were incubated in a 96-well plate for 2 minutes at 50°C, 10 minutes at 95°C, followed by 40 cycles of 15 seconds at 95°C, and 1 minute at 60°C.

Beta-actin and U6 were used as the endogenous controls for relative quantitation analyses for mRNA and miRNA, respectively. Gene expression was analyzed by the $2^{-\triangle\triangle Ct}$ method.

Cell culture

The 293TN, U937, and Kasumi-1 cell lines were incubated at 37 °C in a humidified atmosphere of 5% CO_2 using DMEM or RPMI 1640 media supplemented with 10% (v/v) or 15%(v/v) fetal bovine serum (HyClone, Logan, UT, USA).

Lentivector construction, packaging, and cell infection.

Four siRNAs were designed and synthesized for *FAMLF-1* gene silencing according to the sequence of *FAMLF-1* mRNA, their sequences information were shown in Supplementary Table 5. In addition, siRNA molecules that exhibited no homology to the human genome sequence were selected as negative controls also shown in Online Supplementary Table 5.

Target siRNA fragments were ligated with the transfer vector plasmid pGV248. The primers used for construction, reverse transcription (RT), and PCR are summarized in Online Supplementary Table 3. Two micrograms of every constructed plasmid were co-transfected into 293TN cells using Lipofectamine2000 mixture (Life, Mountain View, CA, USA) according to the manufacturer's instructions. The lentivirus-containing supernatants were collected, filtered, and concentrated by PEG precipitation at 48 h after transfection. To establish cells that stably downregulated the *FAMLF-1* expression, U937 or Kasumi-1 cells were infected with respective lentivirus and then screened by puromycin (final concentration 0.5 ug / ml) at least 72 h after infection for screening stable infected cell lines. The infected cells were collected for detecting the *FAMLF-1* expression to screen best effects of gene silencing between 4 different siRNA group by real-time quantitative RT-PCR. The screened U937 or Kasumi-1 of *FAMLF-1* stable knockdown cells were selected for further function change analysis. A further untreated group of U937 or Kasumi-1 cell cultures served as untreated control group.

Cell proliferation assay

The U937 and Kasumi-1 cells were inoculated at 7.5 × 10^3 cells per well in 96-well plates. To assess the effects of *FAMLF-1* gene silencing on cell proliferation, the *FAMLF-1* stable knockdown cells along with their CON and NC group cells were quantified using CCK-8 assay, according to the manufacturer's instructions. In brief, the cells were cultured in a 96-well plates at a density of 7.5 × 10^3 cells per well. At various time-points of 0, 1, 2, 3, and 4 days, 10 ul of CCK-8 (Dojindo, kamimashiki gun Kumamoto, Japan) solution reagent was added to 100 ul of culture medium in each well. After incubation for 2.5 h at 37°C, The absorbance of each well was read at a double wavelength of 450 and 630 nm on a microplate reader. The measurements were represented by the means of at least three independent experiments, with each data point based on three replicates.

Colony formation assay (methyl cellulose method)

U937 and Kasumi-1 cells of *FAMLF-1* stable knockdown, their CON and NC group cells were collected and counted, and then replated in a 6-well plate at a concentration of 200 cells per well with a final concentration of 0.8% methyl. These samples were then cultured for 14 days in order to form colonies. Images of the colonies were then captured under a inverted fluorescence microscope. Finally, the total number of colonies (cell number more than 50) was counted and analyzed. The measurements were performed by at least three independent experiments, with each data based on three replicates.

Cell cycle analysis

The cell cycle parameters for U937 and Kasumi-1 cells of *FAMLF-1* stable knockdown, their CON and NC group cells were determined by flow cytometry of

propidium iodide stained. In brief, 2 × 10^6 cells of each group were washed in PBS and fixed in 70% ethanol at 4°C overnight. Then fixed cells were centrifuged, washed, and resuspended in PBS containing RNase A and propidium iodide (50 μg/mL each) and incubated for 30 minutes at 37°C for 30 min in dark. Cell cycle distribution was measured on a BD Biosciences FACScan. The percentage of cells present in G0/G1, S and G2/M phases was analysised using the ModFit software package. The measurements were performed by at least three independent experiments.

Assessment of apoptosis

FITC Annexin V apoptosis determining kit was used to quantitatively determine the percentage of cells within a population which were actively undergoing early apoptosis according to manufacture's instruction. Apoptosis analysis was measured on a BD Biosciences FACScan. Briefly, the cells were collected, washed twice with Annexin binding buffer and incubated with Annexin V-PE and 7-AAD under the manufacturer's recommended conditions. Cells that stain positive for FITC Annexin V-PE and negative for 7-AAD were considered undergoing early apoptosis. The measurements were repeated at lease three times independently.

Differentiation determination

The specific markers for myeloid differentiation were detected by flow cytometry. Birefly, every group cells as above were immunostained with 20 ul of CD11b-PE, CD13-APC, CD33-PE, CD34-PerCP antibodies (CST, Danvers, MA, USA) and analyzed on a BD Biosciences FACScan. The measurements were repeated at lease three times independently.

Western blotting

Equal amounts (80 ug) of total protein samples were resolved by SDS-PAGE on 10% gels. Proteins were probed using rabbit anti-AKT-t, anti-AKT-p, anti-c-Myc, and anti-CDK, and anti-actin polyclonal antibodies (CST, Danvers, MA, USA), then incubated with the corresponding horseradish peroxidase-conjugated secondary antibodies (Thermo Scientific, Rockford, IL, USA). Proteins were visualized by using a chemiluminescence detection system (Amersham Biosciences, Piscataway,USA). The experiments were repeated at lease three times independently.

Statistical methods and analysis

Quantitative variables were described in the form of median and interquartile range. Qualitative variables were described as number and percentage. Kruskal-Wallis H and Nemenyi test were used to analyze differential expression of *FAMLF-1* among the two groups (the AML group and the healthy control group). Mann-Whitney *U* test was used to analyze the expression of *FAMLF-1* between PBMCs from the patients with various AML subtypes and those from healthy controls. Additionally, using this test, the expression of miR-181a between AML patients and healthy controls was analyzed. Mann-Whitney *U* test (comparison between two groups), Kruskal-Wallis H test (comparison of more than two groups), Spearman correlation and exact chi-square test were used to analyze the relationship between the expression of *FAMLF-1* and clinical hematological features of AML patients and healthy controls. Spearman correlation was also performed to analyze the correlation between *FAMLF-1* and miR-181a expression.

To evaluate the impact of *FAMLF-1* expression levels on clinical outcome, patients were divided into low and high expression groups using the median *FAMLF-1* expression level. Analyses involved the using of cut-off median level was performed between *FAMLF-1* expression and hemoglobin level, percentage of peripheral blood blasts and HBDH level in the AML group. The Kaplan-Meier method was used to calculate estimated probabilities of RFS and OS. Differences between RFS distributions were analyzed using the log-rank test, while that of OS distributions were evaluated using Wilcoxon test.

Univariate logistic regression models were constructed to evaluate *FAMLF-1* expression for achievement of CR, and univariate Cox proportional hazards models were used to evaluate the associations of *FAMLF-1* expression with RFS and OS. Of the factors in univariate models, those significant at $P \leq 0.2$ were used in a limited backward selection procedure to build multivariable models by retaining the main variable *FAMLF-1* through model building. Thus, multivariable logistic regression models were constructed to analyze factors related to the probability of achieving CR, and multivariable Cox proportional hazards models were constructed to analyze factors important for RFS and OS.

All data were analyzed with SPSS version 18.0 software (SPSS Inc., Chicago, IL, USA). Continuous variables were expressed as median (min-max). Comparisons were made using unpaired *t*-test or nonparametric Mann-Whitney U test and Kruskal-Wallis test for continuous variables and χ^2 test for categorical variables between groups. Correlations between continuous variables were calculated using the Spearman correlation coefficient.

Patients with rare recurrent cytogenetic aberrations such as t(7;12)(q36;p16), t(6;9)(p23;q34), t(16;21)(p11;q22), t(8;16)(p11;p13), monosomy 7, trisomy 8, and complex karyotype were classified as 1 cytogenetic subgroup entitled "rare cytogenetics."

Supplementary Table 1: Clinical presentation of sequenced members in AML pedigree. See_Supplementary_Table 1.

Supplementary Table 2: Correlation between FAMLF-1 mRNA and clinical parameters of patients in 46 FAB-M2, 86 FAB non-M2 and 55 FAB-M5 subtype. See_Supplementary_Table 2.

Supplementary Table 3: PCR primers for genotyping of *FAMLF* gene family

Polymorphisms	Primer sequence (5′–3′)	Fragment sizes (bp)
Exon 1	Forward: GGCCAATAGGATAGCTCTGT<br>Reverse: CCTGCCAGCTGAATAACCTT	408
Exon 2-1	Forward: TCTGCGACCAAATTAGTAGC<br>Reverse: AGATGCTTATTACAAGTGCC	604
Exon 2-2	Forward: CACATCACTTAGGCGAACCA<br>Reverse: TTCACTGTCAGACGCAGAAG	710
Exon 2-3	Forward: AATCAGCTGTTGGAGTCAGG<br>Reverse: GCCAACAGCACTTATAGACA	698
Exon 2-4	Forward: CAGAATCTCAGAGCTGGTAG<br>Reverse: CCAATAATAGGATCTAGCTG	686
Exon 3	Forward: GAGCTAACACAAGGCACCTA<br>Reverse: TTATTCAGTTGAATTATCAC	480
Exon 4	Forward: ATCGTCTCTAGATTACTTAT<br>Reverse: AAGACAGTTATTGGTTGATT	990
Intronic miR181a1/b1	Forward: TATATGACTAAAGGTACTGT<br>Reverse: ATAGAGAAGCATTATTCTTG	534

Supplementary Table 4: Reaction system of *FAMLF* gene family genotyping

Reagents	Volume (μl)
TakaRa Taq(5 u/ul)	0.5
10×PCR Buffer(Mg2+ plus)	10
dNTP Mixture(each 25 mM)	4
DNA template	4
Forward primer (20 μM)	1
Reverse primer (20 μM)	1
RNase-free water	29.5
Total volume	50

Supplementary Table 5: The siRNAs sequences to knockdowm *FAMLF-1*

Target site	Sequence of siRNA	GC%	Position of FAMLF-1
FAMLF-1 -721	GGACCTAACCAAACAACATAC	42.86%	5′UTR of ORF
FAMLF-1-953	GCTGTCTACTGTTCTCTATGT	42.86%	5′UTR of ORF
FAMLF-1-1548	GGTGTCAGGAATATTTGTAGA	38.10%	Coding sequence of ORF
FAMLF-1-1813	GCCATTTAGCTTGTCTATAAG	38.10%	3′UTR of ORF
NC	TTCTCCGAACGTGTCACGT	52.63%	

Supplementary Table 6: Distribution of Genotypes and Haplotypes in *FAMLF* Gene among the AML pedigree members

Polymorphism	Family members *N* = 13 (%)	Control *N* = 65 (%)	OR	95 % CI	*P*
SNP1					
G allele	0.500	0.8231	4.6522	1.9085–11.3404	0.0004
A/A	2 (0.154)	4 (0.0615)			
A/G	9 (0.692)	15 (0.2308)			
G/G	2 (0.154)	46 (0.7077)			
					0.0009
SNP2					
T allele	0.462	0.7538	3.5729	1.4996–8.5130	0.0029
C/C	2 (0.154)	5 (0.0769)			
C/T	10 (0.769)	22 (0.3385)			
T/T	1 (0.077)	38 (0.5846)			
					0.0037
SNP3					
A allele	0.462	0.7538	0.2799	0.1175–0.6669	0.0029
C/C	2 (0.154)	5 (0.0769)			
C/A	10 (0.769)	22 (0.3385)			
A/A	1 (0.077)	38 (0.5846)			
					0.0037
SNP4					
A allele	0.577	0.600	0.9091	0.3872–2.1344	0.8267
G/G	0 (0.000)	7 (0.1077)			
A/G	11 (0.846)	38 (0.5846)			
A/A	2 (0.154)	20 (0.3077)			
					0.1749
SNP5					
T allele	0.731	0.5615	0.4718	0.1856–1.1998	0.1093
G/G	0 (0.000)	12 (0.1846)			
G/T	7 (0.538)	33 (0.5077)			
T/T	6 (0.462)	20 (0.3077)			
					0.2031
Haplotypes					
ACCA T	0.456	0.0535	14.147	4.771–41.954	2.21e-008
ACCGG	0.044	0.1070	0.361	0.051–2.571	0.2904
GCCAG [a]	0.000	0.000	—	—	—
GCC AT [a]	0.038	0.0392	0.930	0.146–5.930	0.8122
GCCGG [a]	0.000	0.0300	—	—	—
GTA AG [a]	0.000	0.030	—	—	0.3594
GTA AT	0.082	0.4612	0.096	0.023–0.405	0.0002
GTAGG	0.226	0.2553	0.798	0.293–2.173	0.6581
GTAGT [a]	—	—	—	—	—
ACCAG [a]	0.000	0.017	—	—	—

Abbreviations: OR, odds ratio; 95% CI, 95% confidence interval.[*]P<0.05 the difference was statistically significant.
[a]Frequency is less than 0.03.

Supplementary Table 7: General information of AML patients and healthy controls for *FAMLF* gene family sequence analysis

	AML	FAB-M2 patients	FAB non-M2 patients	Healthy controls
Case	93	33	60	65
Age (years)[a]	45(30–57)	52(36–60)	38 (25–52)	30 (19–63)
Sex				
Male	50(53.76%)	17(51.52%)	33 (55%)	38 (%)
Female	43(46.24%)	16 (48.48%)	27 (45%)	27 (%)
WBC ($\times 10^9$/L)[a]	25.28 (6.99–63.14)	21.94 (6.5–61.75)	31.47 (10–68.66)	6.98 (4.12–9.25)
Hemoglobin (g/L)[a]	72 (60–84)	74 (54–94)	71 (60–79)	135 (106–159)
PLT ($\times 10^9$/L)[a]	37 (20–67)	48 (26–71)	28 (29–60)	197 (118–372)
BLAST (%)[a]	64 (27–84)	76 (27–83)	60 (28–84)	

[a]Data were expressed as median (min-max) or percentage as indicated. P values were calculated by using unpaired t-test for age, and by χ^2 test for gender percentage. There were not significant difference between AML, FAB-M2, FAB non-M2 group and healthy volunteers in all characteristics ($P > 0.05$).

Supplementary Table 8: Distribution of Genotypes and Haplotypes in *FAMLF* Gene and Associations with Risk of AML and M2 patients

Polymorphism	AML patients					FAB-M2 patients				
	AML patients *N* = 93 (%)	Control *N* = 65 (%)	OR	95 % CI	*P*	FAB-M2 patients *N* = 33 (%)	Control *N* = 65 (%)	OR	95 % CI	*P*
SNP1										
G allele	0.8118	0.8231	1.0783	0.6029–1.9288	0.7994	0.8485	0.8231	0.8307	0.3697–1.8670	0.6533
A/A	6 (0.0645)	4 (0.0615)				4 (0.1212)	4 (0.0615)			
A/G	23 (0.2473)	15 (0.2308)				2 (0.0606)	15 (0.2308)			
G/G	64 (0.6882)	46 (0.70 77)				27 (0.8182)	46 (0.7077)			
					0.9657					0.0836
SNP2										
T allele	0.7473	0.7538	1.0355	0.6167–1.7388	0.8950	0.8030	0.7538	0.7512	0.3634–1.5527	0.4391
C/C	9 (0.0968)	5 (0.0769)				4 (0.1212)	5 (0.0769)			
C/T	29 (0.3118)	22 (0.3385)				5 (0.1515)	22 (0.3385)			
T/T	55 (0.5914)	38 (0.5846)				24 (0.7273)	38 (0.5846)			
					0.8793					0.1390
SNP3										
A allele	0.7473	0.7538	0.9657	0.5751–1.6216	0.8950	0.8030	0.7538	1.3312	0.6440–2.7517	0.4391
C/C	9 (0.0968)	5 (0.0769)				4 (0.1212)	5 (0.0769)			
C/A	29 (0.3118)	22 (0.3385)				5 (0.1515)	22 (0.3385)			
A/A	55 (0.5914)	38 (0.5846)				24 (0.7273)	38 (0.5846)			
					0.8793					0.1390
SNP4										
A allele	0.5108	0.6000	0.6960	0.4422–1.0955	0.1168	0.4545	0.6000	0.5556	0.3054–1.0105	0.0531
G/G	23 (0.2473)	7 (0.1077)				15 (0.4545)	7 (0.1077)			
A/G	45 (0.4839)	38 (0.5846)				6 (0.1818)	38 (0.5846)			
A/A	25 (0.2688)	20 (0.3077)				12 (0.3636)	20 (0.3077)			
					0.0877					< 0.001*
SNP5										
T allele	0.5215	0.5615	1.1751	0.7490–1.8435	0.4825	0.5000	0.5615	1.2807	0.7071–2.3196	0.4139
G/G	22 (0.2366)	12 (0.1846)				13 (0.3939)	12 (0.1846)			
G/T	45 (0.4839)	33 (0.5077)				7 (0.2121)	33 (0.5077)			
T/T	26 (0.2796)	20 (0.3077)				13 (0.3939)	20 (0.3077)			
					0.7307					0.0116*
Haplotypes										
ACCA T	0.0644	0.0535	1.1482	0.4488–3.0855	0.7405	0.0606	0.0534	1.0952	0.3080–3.8945	0.8882
ACCGG	0.1130	0.1070	1.0102	0.4984–2.1024	0.9492	0.0606	0.1070	0.5140	0.1619–1.6320	0.2519
ACCGT [a]	—	—	—	—	—	0.0303	0.0000	0.0000	0.0000–0.0000	0.0505
GCC AT	0.0411	0.0392	0.9856	0.3229–3.1862	0.9805	0.0152	0.0392	0.3612	0.0414–3.1474	0.3371
GCCGG [a]	0.0234	0.0300	0.7477	0.1869–2.9912	0.6801	0.0303	0.0300	0.9697	0.1716–5.4797	0.9722
GTA AG [a]	—	—	—	—	—	—	—	—	—	—
GTA AT	0.3998	0.4612	0.7529	0.4584–1.1521	0.1740	0.3788	0.4612	0.6573	0.3564–1.2123	0.1781
GTAGG	0.3367	0.2553	1.3874	0.8595–2.3460	0.1702	0.4091	0.2553	**1.9230**	1.0201–3.6250	0.0419*
GTAGT [a]	—	—	—	—	—	—	—	—	—	—
ACCAG [a]	—	—	—	—	—	—	—	—	—	—

Abbreviations: OR, odds ratio; 95% CI, 95% confidence interval. .*P<0.05 the difference was statistically significant.

[a] Frequency is less than 0.03.

Supplementary Table 9: Distribution of Genotypes and Haplotypes in *FAMLF* Gene and Associations with Risk of FAB non-M2 and FAB-M5 patients

Polymorphism	FAB non-M2 patients					FAB-M5 patients				
	FAB non-M2 patients *N* = 60(%)	Control *N* = 65 (%)	OR	95 % CI	*P*	FAB-M5 patients *N* = 41(%)	Control *N* = 65 (%)	OR	95 % CI	*P*
SNP1										
G allele	0.7917	0.8231	1.2243	0.6520–2.2989	0.5288	0.7805	0.8231	1.3084	0.6561–2.6091	0.4445
A/A	2 (0.0333)	4 (0.0615)				1 (0.0244)	4 (0.0615)			
A/G	21 (0.3500)	15 (0.2308)				16 (0.3902)	15 (0.2308)			
G/G	37 (0.6167)	46 (0.7077)				24 (0.5854)	46 (0.7077)			
					0.2944					0.1746
SNP2										
T allele	0.7167	0.7538	1.2108	0.6896–2.1259	0.5053	0.7073	0.7538	1.2672	0.6811–2.3579	0.4543
C/C	5 (0.0833)	5 (0.0769)				3 (0.0732)	5 (0.0769)			
C/T	24 (0.4000)	22 (0.3385)				18 (0.4390)	22 (0.3385)			
T/T	31 (0.5167)	38 (0.5846)				20 (0.4878)	38 (0.5846)			
					0.7416					0.5744
SNP3										
A allele			0.8259	0.4704–1.4502	0.5053	0.7073	0.7538	0.7891	0.4241–1.4683	0.4543
C/C	5 (0.0833)	5 (0.0769)				3 (0.0732)	5 (0.0769)			
A/C	24 (0.4000)	22 (0.3385)				18 (0.4390)	22 (0.3385)			
A/A	31 (0.5167)	38 (0.5846)				20 (0.4878)	38 (0.5846)			
					0.7416					0.5744
SNP4										
A allele	0.5417	0.6000	0.7879	0.4769–1.3017	0.3517	0.5366	0.6000	0.7719	0.4417–1.3489	0.3630
A/A	13 (0.2167)	20 (0.3077)				9 (0.2195)	20 (0.3077)			
A/G	39 (0.6500)	38 (0.5846)				26 (0.6341)	38 (0.5846)			
G/G	8 (0.1333)	7 (0.1077)				6 (0.1463)	7 (0.1077)			
					0.5050					0.5704
SNP5										
T allele	0.5333	0.5615	1.1206	0.6806–1.8451	0.6544	0.5366	0.5615	1.1061	0.6347–1.9274	0.7220
G/G	9 (0.1500)	12 (0.1846)				6 (0.1463)	12 (0.1846)			
G/T	38 (0.6333)	33 (0.5077)				26 (0.6341)	33 (0.5077)			
T/T	13 (0.2167)	20 (0.3077)				9 (0.2195)	20 (0.3077)			
					0.3555					0.4375
Haplotypes										
ACCAT	0.0484	0.0535	0.8561	0.2760–2.6562	0.7879	0.0375	0.0535	0.6511	0.1651–2.5681	0.5372
ACCGG	0.1600	0.1070	1.5080	0.7184–3.1656	0.2755	0.1820	0.1070	1.7442	0.7906, 3.8478	0.1648
GCCAT	0.0750	0.0392	1.8914	0.6190–5.7794	0.2567	0.0732	0.0392	1.8251	0.5414, 6.1521	0.3258
GCCGG	0.0000	0.0300	0.0000	0.0000–0.0000	0.0502	0.0000	0.0300	0.0000	0.0000,0.0000	0.1036
GTAAG[a]	—	—	—	—	—	—	—	—	—	—
GTA AT	0.4100	0.4612	0.7412	0.4460–1.2320	0.2476	0.4259	0.4612	0.7802	0.4444, 1.3695	0.3869
GTAGG	0.2984	0.2553	1.1647	0.6662–2.0361	0.5926	0.2814	0.2553	1.0599	0.5675, 1.9796	0.8551
ACCAG[a]	—	—	—	—	—	—	—	—	—	—
GTAGT[a]	—	—	—	—	—	—	—	—	—	—

Abbreviations: OR, odds ratio; 95% CI, 95% confidence interval.

[a] Frequency was less than 0.03.

Supplementary Table 10: The expression of *FAMLF-1* in U937 and Kasumi-1 cells after *FAMLF-1*-RNAi-LV infection

Cell line	Group	Gene	Mean of CT	ΔCt	ΔΔCt	$2^{-\Delta\Delta C t}$	FAMLF-1 inhibition ratio (%)
U937	CON	*FAMLF-1*	18.450	4.983	0	1	0
		AC	13.467				
	NC	*FAMLF-1*	18.501	4.989	0.007	0.996	0
		AC	13.512				
	721	*FAMLF-1*	18.798	5.269	0.286	0.820	18
		AC	13.530				
	953	*FAMLF-1*	18.699	5.211	0.228	0.854	15
		AC	13.488				
	1813	*FAMLF-1*	19.751	6.184	1.202	0.435	57
		AC	13.567				
	1548	*FAMLF-1*	20.512	6.925	1.942	0.260	74
		AC	13.587				
Kasumi-1	NC	*FAMLF-1*	24.538	10.277	0	1	0
		AC	14.261				
	1548	*FAMLF-1*	27.278	12.842	2.565	0.169	83
		AC	14.436				

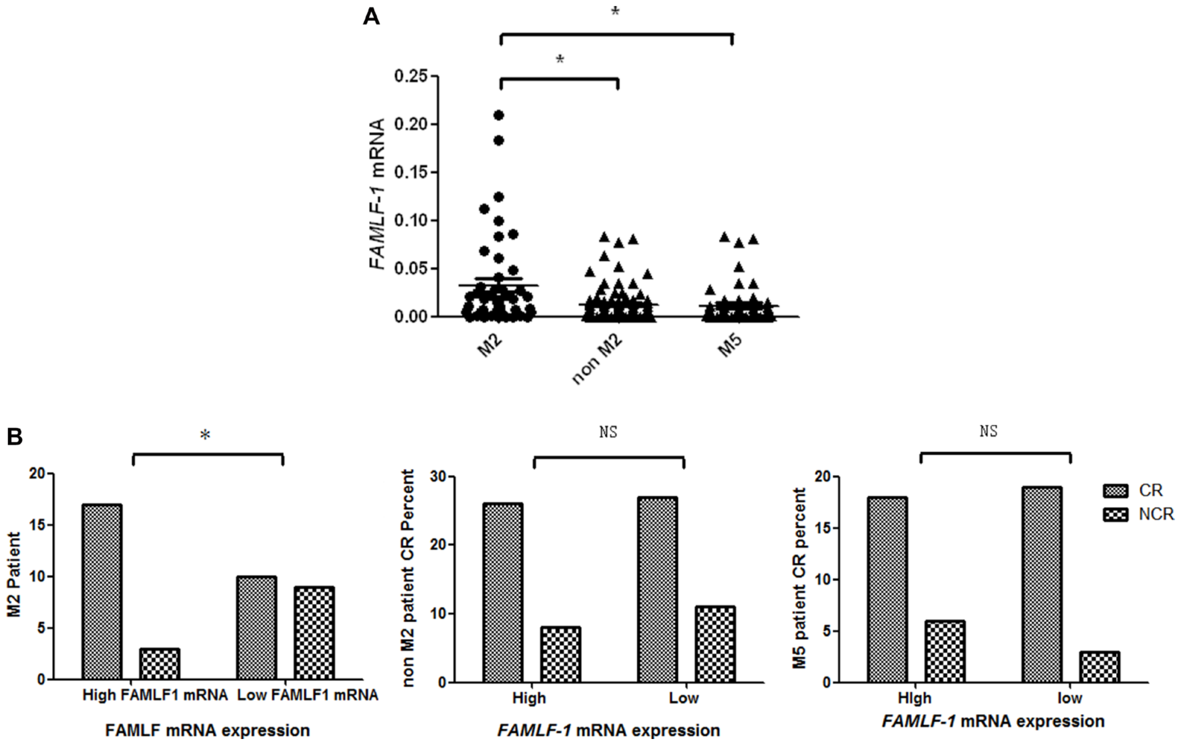

Supplementary Figure 1: The expression levels of FAMLF-1 and CR rate in patients with FAB-M2 and FAB non-M2, FAB-M5. (**A**) The expression levels of FAMLF-1 in patients with FAB-M2 and FAB non-M2, FAB-M5 ($^{**}P = 0.0152$, $^{*}P = 0.0078$). (**B**) The relationship between expression levels of FAMLF-1 and CR rate in patients with FAB-M2, FAB non-M2 and FAB-M5 ($^{*}P = 0.0407$).

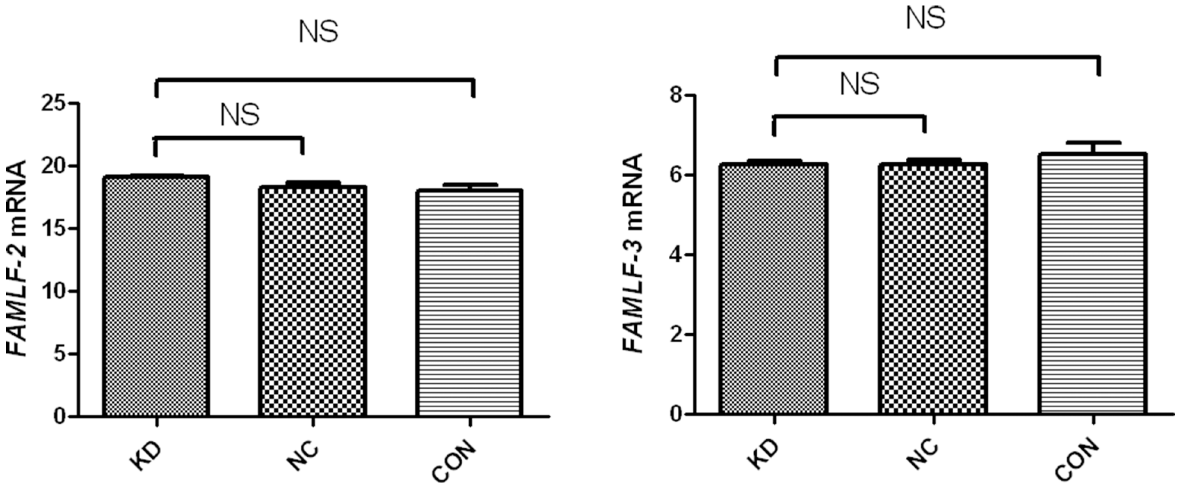

Supplementary Figure 2: FAMLF-2 and FAMLF-3 expression levels changes of Kasumi-1 cells after FAMLF-1 gene silencing($^{*}P > 0.05$).

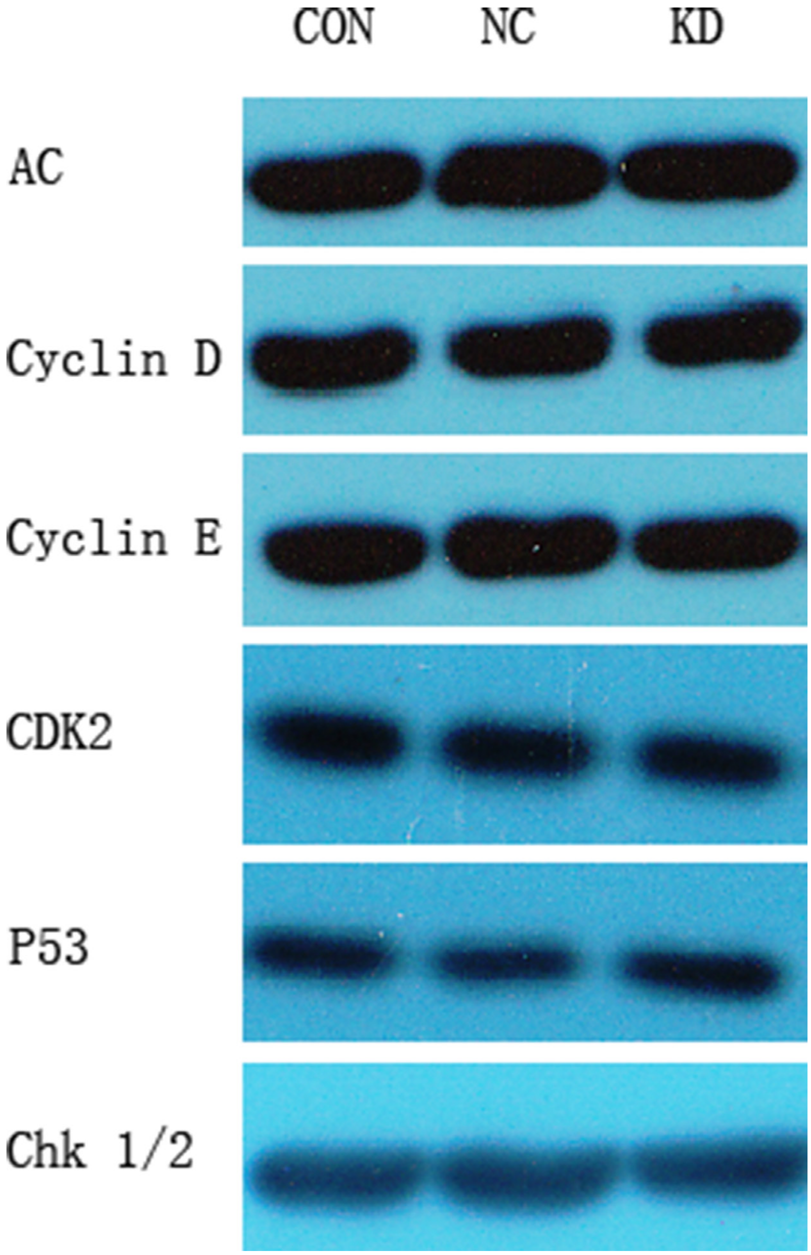

Supplementary Figure 3: Cyclin D、Cyclin E、CDK2、 P53 and Chk 1/2 protein expression changes of the Kasumi-1 cells after FAMLF-1 gene silencing. We found that Cyclin D, Cyclin E, CDK2, P53 and Chk 1/2 associated with cell cycle G1/S arrested and cell proliferation inhibition were no significant change after FAMLF-1 gene silencing.
