# Supplementary material for: Lentivirus-mediated RNA interference targeting FAMLF-1 inhibits cell growth and enhances cell differentiation of acute myeloid leukemia partially differentiated cells via inhibition of AKT and c-MYC
Source: Oncotarget. 2017 Sep 26;8(60):101372–82. doi: 10.18632/oncotarget.21276 (PMC5731881; doi:10.18632/oncotarget.21276)
Supplement: Supplementary file 2 [file oncotarget-08-101372-s002.docx]

Supplementary Table 1: Clinical presentation of sequenced members in AML pedigree.

|  |  |  | Blood count(median) | | |  |  |  |  |
| --- | --- | --- | --- | --- | --- | --- | --- | --- | --- |
|  |  |  | WBC | Plt | Hb | Bone marrow aspirate | Karyotype | Clinical features | Vital status  (age at last  follow-up,  years) |
| ID | Sex | Age | 10^9^/l | 10^9^/l | g/dl |  |  |  |  |
| IV19 | M | 13 | 58 | 20 | 63 | Hypercellular, with 78% myeloperoxidase-positive blast cells,diagnosed with FAB-M2 in February 2003. , | Unidentified | He was treated with daunorubicin and cytarabine and entered a complete remission. He had a relapse within 3 months, never entered a second remission and died in November 2003 | Dead(13) |
| III15 | M | 37 | 69 | 45 | 65 | Hypercellular, with 91% myeloperoxidase-positive blast cells,diagnosed with AML-M1 in March 2008. | 2007: 46,XY; 2008 (after onset):  45,X,-Y. | He rejected chemotherapy for  financial reasons, and died  within 2 months | Dead (37) |
| III5 | F | 43 | 5.1 | 213 | 85 | Hypercellular; 3% erythroid precursors with dysplastic features, including megaloblastic changes, and mature red cells with anisocytosis. | The frequency of chromosome breakage events was increased 18% by DNA cross-linking agents exposure. | A long history of paleness and easy fatigue, and can not be explained by any underlying medical condition. | Alive(47) |
| III14 | M | 44 | 4.5 | 72 | 102 | Hypercellular; 4.5% megakaryocytes with dysplastic features, including micromegakaryocytes and multinucleated megakaryocytes | The frequency of chromosome  breakage events was increased 4% by  DNA cross-linking agents exposure. | A long history of easy bruising and frequent epistaxis. | Alive(48) |
| III8 | M | 60 | 3 | 153 | 132 | Normocellular, 3.5% granulocytes with dysplastic features, including agranular myelocytes, hypersegmented or polyploidy neutrophils. | Unidentiﬁed. | He was an obligate carrier for that his daughter died with AML. He did not show any symptoms at last follow-up. | Alive(64) |
| IV10 | M | 35 | 5.2 | 127 | 138 | Hypercellular, with 4% myeloblasts. | 2007:46,XXY,þ15,19,21, (2q1,18q–).2008:46,XY,del(11q22). | He did not show any symptoms. | Alive (39) |
| IV7 | F | 41 | 13.1 | 87 | 101 | Hypercellular; 3% granulocytes with dysplastic features, including  agranular myelocytes and hypersegmented neutrophils; 3% erythroid precursors with dysplastic features, including nuclear atypia and some megaloblastic changes. | The frequency of chromosome breakage events was increased 8% by DNA cross-linking agents exposure. | A long history of gingival bleeding, increased menstrual and paleness. Her mother died of AML. | Alive (45) |
| IV13 | F | 26 | 14.1 | 224 | 80 | Hypercellular; 3% erythroid precursors with dysplastic features. | 46, XX, 19qhþ. | A history of unexplained recurrent miscarriage. | Alive (30) |
| III13 | M | 50 | N | N | N | N | 46,XY | None. | Alive (54) |
| III8’s wife | F | 55 | N | N | N | N | 46,XX. | None. | Alive (59) |
| V3 | F | 11 | N | N | N | N | 46,XX. | None. | Alive (15) |
| III15’s wife | F | 35 | N | N | N | N | 46,XX. | None. | Alive (39) |
| IV18 | F | 27 | N | N | N | N | 46,XX. | None. | Alive (31) |

Abbreviations: N, normal; None, no haematological manifestations.

The cytopenia of family members had been pre-excluded of iron-deficiency anaemia and megaloblastic anaemia.^[12]^
