# Supplementary material for: Lentivirus-mediated RNA interference targeting FAMLF-1 inhibits cell growth and enhances cell differentiation of acute myeloid leukemia partially differentiated cells via inhibition of AKT and c-MYC
Source: Oncotarget. 2017 Sep 26;8(60):101372–82. doi: 10.18632/oncotarget.21276 (PMC5731881; doi:10.18632/oncotarget.21276)
Supplement: Supplementary file 3 [file oncotarget-08-101372-s003.docx]

**Supplementary Table 2：Correlation between *FAMLF-1* mRNA and clinical parameters of patients in 46 FAB-M2, 86 FAB non-M2 and 55 FAB-M5 subtype**

|  | **FAB-M2(n=46)** | | **FAB non-M2(n=86)** | | **FAB-M5(n=55)** | |
| --- | --- | --- | --- | --- | --- | --- |
|  | ***FAMLF-1* mRNA** | ***P*-level** | ***FAMLF-1* mRNA** | ***P*-level** | ***FAMLF-1* mRNA** | ***P*-level** |
| **Age, years** |  | *P*=0.884 ^b^ |  | *P*=0.7381 ^b^ |  | *P*=0.8358^b^ |
| <60 | 0.03082(0.0009-0.0311) (n=35) |  | 0.0052(0.0007-0.0165) (n=68) |  | 0.0041(0.0004-0.0120) (n=45) |  |
| ≥60 | 0.03794(0.0044-0.0283) (n=11) |  | 0.0049(0.0006-0.0150) (n=18) |  | 0.0048(0.0006-0.01149) (n=10) |  |
| **Sex** |  | *P*=0.448^b^ |  | *P*=0.5171 ^b^ |  | *P*=0.1978^b^ |
| Male | 0.02436(0.0005-0.0281) (n=24) |  | 0.0049(0.0004-0.0150) (n=48) |  | 0.0019(0.0002-0.0114) (n=28) |  |
| Female | 0.04110(0.0044-0.0855) (n=22) |  | 0.0065(0.0020-0.0165) (n=38) |  | 0.0053(0.0007-0.0129) (n=27) |  |
| **2 courses of induction chemotherapy**  CR  PR+NR  **Cytogenetics**  Favourable  Intermediate  Adverse  Unknow  **Hemoglobin ( g/L)**  ＜73  ≥73  **WBC count(10^9/L)**  ＜24.75  ≥24.75  **Platelet count(10^12/L)**  ＜34  ≥34  **Perpheral blood blast (%)**  ＜69.5  ≥69.5  **LDH**  ＜409  ≥409  **HBDH**  ＜360  ≥360  **AML1-ETO**  Negative  Positive  **CBFβ/MYH11**  Negative  Positive  **HOX11**  Negative  Positive  **EVI1**  Negative  Positive  **CEBPA**  Wild type  Mutated  **FLT3-ITD**  Wild type  Mutated  **NPM1**  Wild type  Mutated  **C-KIT**  Wild type  Mutated | 0.03936(0.0040-0.0689) (n=21)  0.00772(0.0005-0.0077) (n=18)  0.04288(0.0002-0.0311) (n=8)  0.03902(0.0020-0.0479) (n=17)  0.00876(0.0000-0.0050) (n=7)  0.03007(0.0075-0.0283) (n=14)  0.0171(0.0005-0.0149) (n=23)  0.0477(0.0089-0.0689) (n=23)  0.0150(0.0009-0.0186) (n=25)  0.0531(0.0089-0.0689) (n=21)  0.0184(0.0002-0.0224) (n=19)  0.0422(0.0017-0.0609) (n=27)  0.0076(0.0002-0.0149) (n=22)  0.0551(0.0089-0.0833) (n=24)  0.0250(0.0009-0.0609) (n=23)  0.0397(0.0044-0.0411) (n=23)  0.0151(0.0005-0.0183) (n=22)  0.0214(0.0224-0.0855) (n=24)  0.0356(0.0040-0.0311) (n=39)  0.0144(0.0002-0.0243) (n=7)  0.0268(0.0009-0.0243) (n=40)  (n=0)  0.0255(0.0017-0.0243) (n=38)  0.0515(0.0040-0.0990) (n=2)  0.0337(0.0017-0.0311) (n=44)  0.0035(0.0021-0.0049) (n=2)  0.0201(0.0002-0.0224) (n=21)  0.0750(0.0311-0.0855) (n=4)  0.0307(0.0002-0.0311) (n=23)  0.0076(0.0050-0.0103) (n=2)  0.0296(0.0002-0.0270) (n=24)  0.0110 (n=1)  0.0292(0.0002-0.0270) (n=24)  0.0224(n=1) | *P*=0.044^b^  *P*=0.217^c^  ***P*=0.0011^b^**  ***P*=0.0019^b^**  *P*=0.2743^b^  ***P*<0.0001^b^**  *P*=0.0788^b^  ***P*=0.0025^b^**  *P*=0.4445^b^  _  _  _  *P*=0.2213^b^  _  _  _ | 0.0050(0.0005-0.0170) (n=42)  0.0048(0.0004-0.0129) (n=32)  0.0079(0.0009-0.0248) (n=24)  0.0053(0.0004-0.0150) (n=37)  0.0035(0.0001-0.0129) (n=17)  0.0069(0.0008-0.0069) (n=8)  0.01983(0.0004-0.0129) (n=41)  0.01303(0.0006-0.0150) (n=45)  0.0063(0.0006-0.0237) (n=41)  0.0048(0.0011-0.0129) (n=45)  0.0091(0.0027-0.0170) (n=45)  0.0028(0.0000-0.079) (n=41)  0.01179(0.0004-0.0129) (n=44)  0.02096(0.0019-0.0165) (n=42)  0.0056(0.0007-0.0129) (n=42)  0.0049(0.0004-0.0160) (n=44)  0.0045(0.0007-0.0176) (n=43)  0.0052(0.0011-0.0193) (n=43)  0.0052(0.0007-0.0160) (n=82)  0.0284(0.0045-0.0522) (n=4)  0.0052(0.0006-0.0165) (n=78)  0.0064(0.0001-0.0104) (n=8)  0.0049(0.0005-0.0160) (n=75)  0.0028(0.0037-0.0176) (n=3)  0.0050(0.0007-0.0165) (n=75)  0.0064(0.0006-0.0091) (n=3)  0.0048(0.0011-0.0160) (n=47)  0.0342(0.0019-0.0812) (n=5)  0.0079(0.0011-0.0176) (n=39)  0.0035(0.0002-0.0142) (n=13)  0.0064(0.0011-0.0176) (n=50)  -(0.00004-0.0029) (n=2)  0.0049(0.0011-0.0165) (n=52)  (n=0) | *P*=0.8743^b^  *P*=0.4433^c^  *P*=0.6972^b^  *P*=0.4065^b^  *P*=0.0530^b^  *P*=0.2939 ^b^  *P*=0.9828^b^  *P*=0.6409^b^  *P*=0.5051^b^  *P*=0.6758^b^  *P*=0.8966^b^  *P*=0.8139^b^  *P*=0.2913^b^  *P*=0.8365^b^  _  _ | 0.0029(0.0003-0.0129) (n=37)  0.0048(0.0004-0.090) (n=9)  0.0079(0.0000-0.0522) (n=13)  0.0037(0.0004-0.0115) (n=27)  0.0041(0.0002-0.0047) (n=10)  0.0065(0.0008-0.0065) (n=5)  0.0037(0.0008-0.0079) (n=28)  0.0048(0.0002-0.0062) (n=27)  0.0028(0.0002-0.0176) (n=23)  0.0045(0.0008-0.0114) (n=32)  0.0065(0.0011-0.0129) (n=24)  0.0028(0.0004-0.0079) (n=31)  0.0027(0.0004-0.0079) (n=30)  0.0065(0.0011-0.0165) (n=25)  0.0041(0.0007-0.0115) (n=26)  0.0045(0.0004-0.0129) (n=29)  0.0029(0.0003-0.0114) (n=28)  0.0048(0.0004-0.0115) (n=27)  0.0037(0.0004-0.0129) (n=52)  0.0522(0.0045-0.0836) (n=3)  0.0045(0.0006-0.0129) (n=50)  0.0009(0.0000-0.0079) (n=5)  0.0045(0.0004-0.0129) (n=48)  - (0.0028-0.0037) (n=2)  0.0045(0.0004-0.00129) (n=54)  0.0006(-) (n=1)  0.0045(0.0009-0.0129) (n=29)  0.0342(0.0000-0.0812) (n=5)  0.0079(0.0011-0.0277) (n=26)  0.0045(0.0002-0.0047) (n=9)  0.0047(0.0011-0.0193) (n=32)  -(0.0000-0.0029) (n=2)  0.0047(0.0011-0.0028) (n=34)  - (n=0) | *P*=0.6378^b^  *P*=0.6196^c^  *P*= 0.8333^b^  *P*= 0.9796^b^  *P*=0.2966^b^  *P*= 0.0529^b^  *P*= 0.7680^b^  *P*=0.9396^b^  *P*=0.0666^b^  *P*=0.3267^b^  _  _  *P*=0.3308^b^  *P*=0.4052^b^  _  _ |

^a^Values represent median and range, while other values present as median and interquartile range.

^b^ Mann-Whitney U-test.

^c^Kruskal-Wallis test.

^d^exact chi-square test

^*^ Significant at P < 0.05. The statistically significant P values were displayed in bold font. Scribted “-“ item was due to the small sample size which was not statistically analyzed.
